# Growth stage-specific responses of cucumber to salinity stress: germination, seedling establishment, and vegetative development

**DOI:** 10.3389/fpls.2025.1617809

**Published:** 2025-08-13

**Authors:** Azeezahmed Shaik, Raghupathy Karthikeyan, Chandrasekar S. Kousik

**Affiliations:** ^1^ Oak Ridge Institute for Science and Education (ORISE) Participant Sponsored by the U.S. Department of Agriculture—Agricultural Research Service, U.S. Vegetable Laboratory, Charleston, SC, United States; ^2^ Department of Agricultural Sciences, College of Agriculture, Forestry and Life Sciences, Clemson University, Clemson, SC, United States; ^3^ Coastal Research and Education Center, Clemson University, Charleston, SC, United States; ^4^ U.S. Department of Agriculture—Agricultural Research Service, U.S. Vegetable Laboratory, Charleston, SC, United States

**Keywords:** brackish water, salinity tolerance, cultivar response, germination rate, seedling survival rate, salinity stress indices, gas exchange

## Abstract

Salinity is a major abiotic stress limiting cucumber (*Cucumis sativus* L.) production, especially in areas where saltwater intrusion is present and brackish water is used for irrigation. This study evaluated salinity tolerance in cucumber cultivars across three growth stages—germination, seedling, and vegetative—using various concentrations of brackish water ranging from 0 to 31 dS·m^-1^. Germination results revealed distinct cultivar responses, with ‘Diva’ performing well and ‘Striped Armenian’ showing poor emergence. However, at the seedling and vegetative stages, ‘Striped Armenian’ consistently outperformed others under salinity stress, maintaining higher survival, shoot growth, and stress tolerance indices. In contrast, cultivars such as ‘Diva’ and ‘H-19 Little Leaf’ were more sensitive at later stages despite good early germination. Brackish water concentrations above 6 dS·m^-1^ led to significant reductions in biomass and shoot traits, with complete seedling mortality observed at 22 dS·m^-1^. At the vegetative stage, increasing salinity resulted in reduced shoot length, dry weight, and gas exchange parameters, including stomatal conductance, transpiration, photosynthesis, and intercellular carbon dioxide concentration. While intrinsic water use efficiency increased under severe stress, it did not consistently indicate overall tolerance. The contrasting performance of cultivars across stages underscores the complexity of salinity responses. Cultivar ‘Striped Armenian’ consistent resilience suggests its potential for use in areas where saltwater intrusion is a problem. These findings emphasize the importance of stage-specific screening and the integration of physiological traits to accurately identify salt-tolerant cultivars. This approach provides a reliable strategy for improving cucumber productivity under saline conditions and supports breeding efforts targeting stress-resilient varieties.

## 1 Introduction

Cucumber (*Cucumis sativus* L.), a high-value horticultural crop with global importance, is highly sensitive to salinity stress (Chartzoulakis, 1994; Chen et al., 2020). Cucumber respond differentially to salinity in terms of both morphology and physiology at different stages of growth (Marium et al., 2019; Elsheery et al., 2020). Seed germination and early seedling growth are particularly sensitive stages to salinity stress (Chartzoulakis, 1992; Yildirim et al., 2008; Ibrahim, 2016; Du et al., 2021). During germination, salinity stress disrupts water uptake (Alsaeedi et al., 2019; Bakhshandeh et al., 2021), reduces enzyme activity (Gou et al., 2020; Li et al., 2023a), and inhibits metabolic processes (Zhang et al., 2017; Li et al., 2023a), leading to lower germination rates and weaker seedlings (Li et al., 2023a). Further, salinity limits root and shoot growth and lowers overall vigor during the seedling-establishing phase by causing osmotic stress and ion toxicity (Chen et al., 2020; Al-Momany and Abu-Romman, 2023). Similarly, during vegetative growth, salinity interferes with photosynthesis, stomatal conductance, and water use efficiency, ultimately impairing biomass accumulation and yield (Al-Momany and Abu-Romman, 2023; Amerian et al., 2024). It is important to understand how cucumber responds to salinity at different growth stages to develop tolerant varieties (Huang et al., 2010). However, most studies look at only one stage or a few traits, making it difficult to fully understand salinity tolerance during various growth stages. Furthermore, the only source of salinity stress used in these investigations is usually pure sodium chloride (NaCl). Although studies focused on NaCl offer insightful information, they fall short in capturing the complexities of saline environments with varying water chemistry (Grattan and Grieve, 1998; Munns and Tester, 2008).

Brackish water—a blend of dissolved minerals and salts—can serve as a practical irrigation source in agriculture (Allison, 1964; Rhoades, 1984; Rhoades et al., 1992; Birnhack et al., 2010). When managed properly, it has been shown to support crop production, particularly in salt-tolerant species or under controlled conditions (Grattan et al., 1987; Patel et al., 2003; Sharma and Minhas, 2005). Since crops are vulnerable to salinity in early growth stages, high-quality water is recommended during pre-sowing to improve germination (Rhoades, 1984; Pang et al., 2010). In later stages, diluted brackish water helps maintain salinity within permissible limits, enhancing growth and yield (Abdal and Suleiman, 2003). Studies have demonstrated the benefits of strategic irrigation timing with brackish water. For example, muskmelon (*Cucumis melo* L.) yield under a combined irrigation strategy using freshwater (1.2 dS·m^-1^) and brackish water (7 dS·m^-1^) was comparable to that under freshwater alone, while fruit quality was enhanced under the combined approach (Bustan et al., 2005). Similarly, applying saline water (7.5 dS·m^-1^) at the fourth or eleventh leaf stage in tomato (*Solanum lycopersicum* L.) reduced yield losses to 30%—compared to 70% when applied at earlier stages—while enhancing fruit quality (Pasternak et al., 1986). This emphasizes the importance of strategic timing in brackish water irrigation, showcasing its unique potential. Unlike pure NaCl solutions, brackish water contains a complex mix of ions that interact synergistically or antagonistically, creating distinct stress conditions for plants (Yasuor et al., 2020). These mixed ionic conditions can differentially impact physiological processes such as nutrient uptake, osmotic balance, and membrane stability, depending on the crop species and its developmental stage (Zhang and Du, 2022). However, despite its agricultural relevance, brackish water is underrepresented in salinity research, especially in cucumber.

Cucumber is among the most salt-sensitive horticultural crops (Chartzoulakis, 1992; Lechno et al., 1997; Li et al., 2023b), unlike melons, which are relatively tolerant (Mangal et al., 1988; Shannon and Grieve, 1998), or tomatoes, which show moderate sensitivity (Cuartero et al., 2006). Despite this sensitivity, most cucumber salinity studies focus on NaCl, and little is known about cucumber responses to the more agriculturally relevant brackish water. Brackish water differs fundamentally from NaCl solutions due to its complex ionic composition, which introduces distinct stress pathways involving both beneficial and toxic ions (Grattan and Grieve, 1998). However, current literature provides limited insights into how these mixed-ion profiles affect cucumber physiology and stress adaptation (Chartzoulakis, 1992; Li et al., 2023b). Moreover, plant responses to salinity are not static but vary across developmental stages, and failure to account for stage-specific sensitivities can lead to misleading conclusions. Therefore, understanding cucumber’s growth stage-specific responses to brackish water-induced salinity is essential for developing robust screening protocols and selecting cultivars suitable for saline environments. This study addressed these gaps by evaluating cucumber responses to brackish water salinity at three critical growth stages: germination, seedling establishment, and vegetative development. The specific objectives were to:

1. Evaluate the performance of different cucumber cultivars using various concentrations of locally collected brackish water to identify tolerant cultivars based on germination traits.
2. Assess the tolerance of cucumber cultivars to salinity stress during early seedling growth by measuring survival rates, biomass, and other salinity stress parameters.
3. Examine the effects of brackish water on vegetative growth by analyzing gas exchange traits and shoot characteristics.

## 2 Materials and methods

### 2.1 Brackish water collection

Brackish water was collected from Long Branch Creek, Charleston, South Carolina, USA (32°47’38” N & 80°3’25” W) and its Electrical Conductivity (EC) was measured on-site using a pH/EC meter (Orion STAR A325 pH/conductivity portable meter; Thermo Fisher Scientific, Waltham, MA), which indicated an EC of 40 dS·m^-1^. The collected water was then filtered using a disposable filter (0.2 μm Thermo Scientific™ Nalgene™ Rapid-Flow™) to ensure consistency and remove sediments. Filtered brackish water was used to prepare treatment solutions by diluting it to EC-adjusted concentrations of 3.125% (1.5 dS·m^-1^), 6.25% (3 dS·m^-1^), 12.5% (6 dS·m^-1^), 25% (12 dS·m^-1^), 37.5% (16 dS·m^-1^), 50% (22 dS·m^-1^), and 75% (31 dS·m^-1^). The selected salinity levels (1.5 to 31 dS·m^-1^) span both field-relevant and high-stress conditions. Lower EC levels (1.5 to 6 dS·m^-1^) reflect salinity commonly encountered in vegetable-producing regions of the southeastern U.S. and other marginal water-use zones (USDA, 1997). While higher levels (≥12 dS·m^-1^) were included to assess cultivar tolerance thresholds under extreme brackish conditions typical of arid environments such as the Negev Desert (Bustan et al., 2005). Each diluted concentration was then sent to the Clemson University Agricultural Service Lab (Clemson, South Carolina, USA) for nutrient and salinity analysis across the treatment solutions (**Supplementary Table 1**).

### 2.2 Salinity tolerance in cucumber during germination stage

Two separate germination experiments were conducted to assess the salinity tolerance of cucumber cultivars under controlled conditions. The first experiment (G_1_) was conducted from 1 March to 16 March 2023, while the second experiment (G_2_) was conducted from 24 April to 9 May 2023. In both experiments, temperature (21°C) and relative humidity (40%) were maintained using a Mitsubishi Electronic split air conditioner system (Mitsubishi Electronic Corporation, Tokyo, Japan). In G_1_, 12 cucumber cultivars—Cool Customer, Corinto, Diva, H-19 Little Leaf, Katrina, Lemon, Marketmore 76, Mexican Sour Gherkin, Picolino, Salt and Pepper, Striped Armenian, and Suyo Long (Johnny’s Selected Seeds, Winslow, ME)—were tested across a range of brackish water treatments to evaluate their initial response to salinity. In G_1_, twelve cucumber cultivars were evaluated under seven salinity treatments, which included a deionized water control (0 dS·m^-1^) and six brackish water concentrations: 1.5, 3, 6, 12, 22, and 31 dS·m^-1^. Based on seedling vigor index performance in G_1_, six cultivars—representing the four most vigorous and two least vigorous performers—were selected for G_2_ to capture contrasting responses to salinity. While G_2_ used the same deionized water control as G_1_, it differed by including an additional intermediate salinity level of 16 dS·m^-1^, resulting in a total of eight salinity treatments: 0, 1.5, 3, 6, 12, 16, 22, and 31 dS·m^-1^. This expanded range provided greater resolution for evaluating cultivar responses to moderate-to-high salinity stress during germination. Both experiments were conducted using a completely randomized design (CRD) with four replications for each treatment level.

For each replication, 15 seeds of each cultivar were evenly placed on a 148-mm-diameter sterile blue germination blotter paper (Anchor Paper, St Paul, MN, USA) inside a Nunc (Rochester, NY, USA) Lab-Tek^®^ 150 × 25 mm Petri dishes. A 15 mL of the designated salinity treatment was applied using a multi-dispenser pipette (Repeater^®^ M4, Eppendorf AG, Hamburg, Germany). Germination counts were recorded after seven days to calculate germination percentage. Two weeks after treatment application, additional data were collected using WinRHIZO Pro version 2016a software (Regent Instruments Inc., Quebec, Canada). Cotyledon’s fresh weight was measured using an Ohaus Adventurer model AX324 analytic balance (Ohaus Adventurer ^®^, Parsippany, NJ, USA). Stress tolerance indices were calculated to evaluate the response of cucumber cultivars to salinity stress using the following equations:

$\text{Germination percentage }(\text{GP})=(\frac{\text{number of germinated seeds }}{\text{number of total seeds}})\times 100\;$ (Scott et al., 1984);

$\text{Germination stress tolerance index }=(\frac{\text{GP under salt stress }}{\text{GP under normal control}})\times 100$ (Tarchoun et al., 2022);

Seedling vigor index=(Mean GP×mean seedling length)  (Irik and Bikmaz, 2024);

### 2.3 Salinity tolerance in cucumber during seedling stage

**Figure 1:**
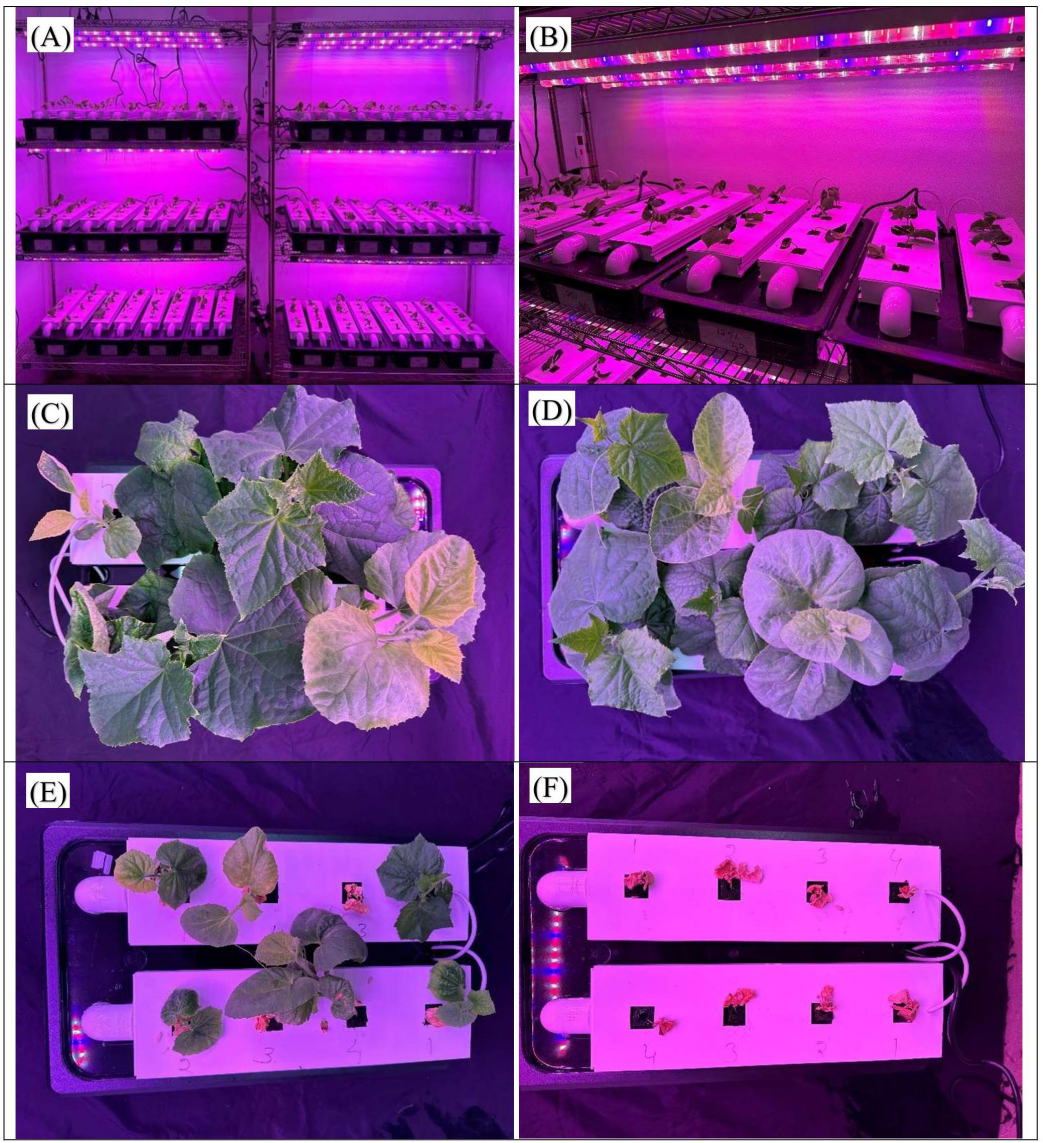
Effect of different brackish water salinity levels on seedling growth of cucumber. **(A)** Experimental setup under a controlled environment. **(B)** Close-up of NFT system used for salinity treatments. Plant performance is shown at different salinity levels: **(C)** 0 dS·m^-1^, **(D)** 6 dS·m^-1^, **(E)** 14 dS·m^-1^, and **(F)** 22 dS·m^-1^.

Four cucumber cultivars—Diva, Katrina, Striped Armenian, and Suyo Long—were selected based on results from the germination experiment for evaluation at the seedling stage. Two experiments were conducted: the first experiment, where seeds were sown on 1 December 2023, and the second experiment, where seeds were sown on 15 January 2024. The seeds were sown in Oasis cubes (OASIS^®^ HORTICUBES^®^, 777 Stow Street Kent, OH, USA), and after 18 days, seedlings were transplanted into a 9 L Nutrient Film Technique (NFT) desktop system (CROPKING^®^, 134 West Drive Lodi, OH, USA). At the time of transplanting, the following treatments were prepared and applied based on the required salinity levels: 0 (control), 6, 12, 14, 16, and 22 dS·m^-1^ of brackish water concentrations. Both experiments were arranged in a completely randomized design and replicated four times (**Figures 1A, B**). Each replication included two seedlings per cultivar per treatment, resulting in a total of 8 plants per cultivar per treatment across the four replications. Twenty-one days after transplanting, data were collected on the following parameters: survival percentage, shoot fresh weight (SFW), shoot length (SL), and chlorophyll content. Chlorophyll content was measured non-destructively using a Soil Plant Analysis Development (SPAD) chlorophyll meter (SPAD-502Plus, Konica Minolta, Tokyo, Japan) from the youngest fully expanded leaf of each plant. Stress tolerance indices were calculated using SFW as measure of plant performance. For each cultivar, data from the control treatment (0 dS·m^-1^) and four salinity treatments (6, 12, 14, and 16 dS·m^-1^) were used to compute the following indices. The highest salinity level (22 dS·m^-1^) was excluded from stress index calculations due to complete mortality in three cultivars; only ‘Striped Armenian’ survived.

$\text{SFW stress tolerance index}=(\frac{\text{SFW under salt stress }}{\text{SFW under control}})\times 100$ (Tarchoun et al., 2022);

$\text{Stress intensity }(\text{SI})=1-(\frac{Y\bar{s}}{Y\bar{p}})$ (Fischer and Maurer, 1978; Ekbic et al., 2017);

$\text{Mean productivity}=\frac{\left(Y_{s}-Y_{p}\right)}{2}$ (Rosielle and Hamblin, 1981; Ekbic et al., 2017);

$\text{Geometric mean productivity}=\;\sqrt{Y_{s\;}\times Y_{p}}$ (Tarchoun et al., 2022);

$\text{Stress tolerance index}=\frac{\left(Y_{s}\times Y_{p}\right)}{{\left(\;Y_{\bar{p}}\right)}^{2}}$ (Tarchoun et al., 2022);

Stress sensitivity index=(1−(YsYp))(1−(Ys¯Yp¯)) (Fischer and Maurer, 1978);

$\text{Tolerance}=(Y_{p}-\;Y_{s})$ (Rosielle and Hamblin, 1981);

Where *Y_p_* represents the performance under non-stress or normal conditions, and *Y_s_* denotes the performance under stress conditions. Additionally, *Y_ $\vec{p}$_* refers to the average performance of the cultivars under normal conditions, while *Y_ $\vec{s}$_* indicates the average performance of the cultivars under stress conditions.

### 2.4 Salinity tolerance in cucumber during vegetative stage

**Figure 2:**
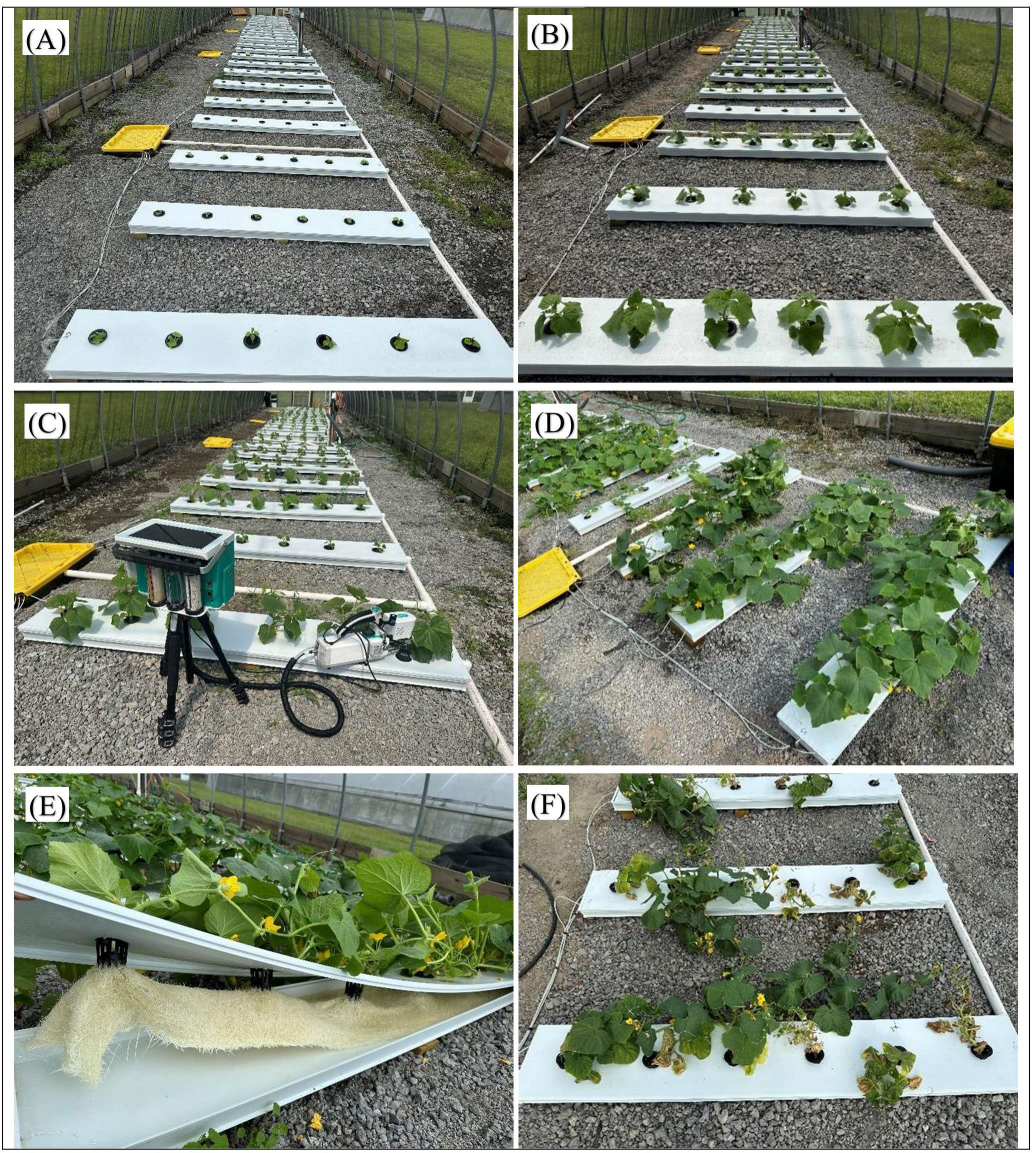
Evaluation of cucumber cultivars response to brackish water salinity under hoophouse conditions. **(A)** Experimental setup at the time of transplanting, prior to salinity treatment initiation. **(B)** Plant status on the day salinity treatment began. **(C)** Gas exchange measurements were conducted 7 days after salinity treatment initiation. **(D)** Plant growth performance under 3 dS·m^-1^ salinity. **(E)** Healthy root development and active nutrient flow observed under 3 dS·m^-1^ salinity. **(F)** Plant performance and visible stress symptoms under 12 dS·m^-1^ salinity at 21 days post salinity treatment.

Based on germination experiment results, six cucumber cultivars— Diva, Katrina, Lemon, H-19 Little Leaf, Suyo Long, and Striped Armenian—were selected for two vegetative-stage experiments conducted in a hoop house. Seeds of the selected cultivars were sown in Oasis cubes (OASIS^®^ HORTICUBES^®^, 777 Stow Street, Kent, OH 44240, USA) on 3 May 2023 for the first experiment and on 15 June 2023 for the second. In both experiments, ten-day-old seedlings were transplanted into 183 cm long × 30 cm wide NFT channels (CROPKING^®^, 134 West Drive, Lodi, OH, USA). Each NFT system was connected to a 102-liter solution tank (Tough Storage Tote, HDX^®^, The Home Depot, 2455 Paces Ferry Road, Atlanta, GA 30339, USA), and the nutrient solution was continuously recirculated using a submersible pump (Active Aqua AAPW400, 370 GPH, Hydrofarm, USA). The pump was fitted with a Venturi air intake system, which included an air valve and muffler assembly positioned above the water surface. This built-in aeration kit induced air into the nutrient stream through the suction side of the pump, ensuring continuous dissolved oxygen delivery without the need for an external air pump. Flow rate was maintained at approximately 1.5 L·min^−^¹, and solution aeration was visible via continuous bubbling within the NFT channels. No active temperature or humidity control was used in the hoop house; however, ambient conditions were monitored using a data logger (HOBO USB Micro Station H21-USB; Onset Computer Corp., Bourne, MA, USA). The average temperature during the experiments was 23.6 °C in May 2023 and 26.0 °C in June 2023, with average relative humidity of 85% and 86%, respectively. Both experiments were set up using a split-plot design with six single-plant replicates per cultivar (**Figure 2A**). During the first 10 days after transplanting, seedlings were supplemented with a 2 dS·m^-1^ nutrient solution (Fertmax Grow A & B, CleanGrow Nutrients, Sebastopol, CA) to promote establishment. On the 11th day after transplanting, salinity treatments (0, 3, 6, and 12 dS·m^-1^) were introduced by mixing brackish water with nutrient solution and adjusted to the designated EC levels (**Figure 2B**). The nutrient solution, along with the respective salinity treatments, was replenished every 5 days to maintain consistent nutrient and salinity conditions throughout the experiment.

Physiological responses of the cultivars to the salinity treatments were assessed at seven days (**Figure 2C**) and 21 days after salinity stress. Four plants from each treatment were sampled, and the following parameters were measured using a portable photosynthesis system (Model LI-6800, LI-COR Biosciences, Lincoln, NE, USA): stomatal conductance, transpiration rate, net photosynthesis rate, intercellular CO_2_ concentration, and intrinsic water use efficiency. Measurements were consistently taken from the same leaf position—young, fully expanded third leaf from the apex—to reduce variability. Prior to data collection, the LI-6800 system was calibrated under steady-state conditions with the following settings: 1,500 µmol·m^−^²·s^−^¹ photosynthetically active radiation (PAR), 400 µmol·mol^−^¹ reference CO_2_ concentration, 700 µmol·s^−^¹ air flow rate, and 65% relative humidity. Temperature control was disabled to reflect ambient conditions (Shaik and Singh, 2022). Each leaf sample was measured for a duration of 2 minutes to ensure stable reading. All measurements were conducted between 10:00 am and 2:00 pm to minimize diurnal variability. In addition to physiological measurements, key growth parameters—including shoot length (SL) and shoot dry weight (SDW)—were also recorded 21 days after salinity stress initiation. For each treatment, four plants per cultivar were selected for growth measurements. These data were used to calculate salinity stress tolerance indices to assess cultivar performance under stress relative to control conditions. The following indices were computed:

$\text{SL stress tolerance index}=(\frac{\text{SL under salt stress }}{\text{SL under control}})\times 100$ (Tarchoun et al., 2022);

$\text{SDW stress tolerance index}=(\frac{\text{SDW under salt stress }}{\text{SDW under control}})\times 100$ (Tarchoun et al., 2022);

These indices provided a normalized measure of vegetative growth performance under salinity stress, with higher values indicating greater tolerance.

### 2.5 Statistical analysis

All data were analyzed using JMP software (version 14.3; SAS Institute Inc., Cary, NC). Statistical analyses were performed separately for each developmental stage—germination, seedling, and vegetative. Each stage included two independent experimental runs, which were initially analyzed separately. Since no significant differences or interactions were detected between runs, data was pooled for final analysis. For each stage, a two-way analysis of variance (ANOVA) was conducted to evaluate the effects of cucumber cultivar, salinity level, and their interaction (cultivar × salinity) on the measured traits. This approach allowed us to determine not only the main effects of cultivar and salinity stress but also whether the cultivars responded differently to varying salinity levels. Before conducting ANOVA, we verified that the assumptions of normality and homogeneity of variance were met using the Shapiro–Wilk test and Levene’s test, respectively. When ANOVA results indicated significant effects (*p* < 0.05), Tukey’s Honestly Significant Difference (HSD) test was used for multiple mean comparisons. Graphs were prepared using SigmaPlot (version 14.5; Systat Software Inc., San Jose, CA).

## 3 Results and discussion

### 3.1 Brackish water composition

The chemical composition of the brackish water used in the study is presented in **Supplementary Table 1**. EC showed a proportional increase with brackish water concentration. It started at 0.009 dS·m^-1^ for the control (0%) and reached 40 dS·m^-1^ at full strength (100%), reflecting a clear gradient of salinity stress. As salinity levels increased, there was a corresponding rise in the concentrations of major ions, including sodium (Na^+^), chloride (Cl⁻), calcium (Ca²^+^), and potassium (K^+^), which play important roles in plant responses to salinity stress (El-Shraiy et al., 2011; Ulas et al., 2020). For example, Na^+^ concentrations increased below detectable limits at 0% to 7,101 ppm at 100% concentration, while (Cl⁻) levels increased from 3 ppm to 4,861 ppm across the same salinity gradient (**Supplementary Table 1**). Similar trends have been reported in other natural brackish water systems, where Na^+^ concentrations reached up to 4,933 ppm and (Cl⁻) up to 2,025 ppm (Mongelli et al., 2013; Nthunya et al., 2018). In contrast, essential micronutrients like zinc (Zn), copper (Cu), and manganese (Mn) remain below detection limits across all treatments. According to Ünlükara et al. (2008), this result aligns with observations that irrigation using brackish water often leads to decreased availability of micronutrients such as Zn, Cu, and Mn due to ionic imbalances and reduced solubility under saline conditions. Interestingly, the pH of the brackish water slightly increased with rising salinity, ranging from 5.8 at 0% to 7.87 at 100% (**Supplementary Table 1**). Total dissolved solids followed a similar trend, increasing from 5 ppm at 0% to nearly 20,000 ppm at 100%, reflecting a substantial salt accumulation (Rhoades et al., 1992). In contrast, the oxidation-reduction potential (mV) decreased with increasing salinity. This suggests a shift in water chemistry and redox conditions, likely driven by elevated ionic strength and reduced oxygen availability (Grattan and Grieve, 1998). These differences in ion composition across salinity levels provide valuable information about the specific ionic stresses experienced by cucumber plants. Unlike studies using pure NaCl, natural brackish water introduces complex ion interactions (Wang et al., 2023). These interactions may differentially affect osmotic and ionic stress responses across growth stages and cultivars (Ludwiczak et al., 2021; Liang et al., 2023). While this study focuses exclusively on brackish water to simulate realistic agricultural conditions, the absence of a NaCl only control limits the ability to fully distinguish osmotic effects from specific ion toxicity or micronutrient imbalances. Nevertheless, the findings offer valuable insights into cultivar performance under practical, field-relevant salinity scenarios.

### 3.2 Germination responses to salinity stress

Germination traits were evaluated across two experiments: G_1_ (initial screening of 12 cucumber cultivars) and G_2_ (a subset of 6 cultivars selected based on G_1_ performance). Salinity level, cultivar, and their interaction significantly (*p* < 0.0001) affected germination percentage, salinity tolerance index, seedling vigor index, and cotyledon fresh weight in both G_1_ and G_2_ (**Table 1**).

**Table 1:** Analysis of variance (ANOVA) and coefficient of variation (CV%) for germination percentage, salinity tolerance index, cotyledon fresh weight, and seedling vigor index in cucumber cultivars under salinity stress conditions during two independent germination experiments (G_1_ and G_2_).

Growth parameter	Source	Germination experiment-1 (G_1_)			Germination experiment-1 (G_2_)		
		F-value	p-value	CV (%)	F-value	p-value	CV (%)
Germination percentage	Salinity (S)	4498.5	<0.0001	5.3	347.6	<0.0001	12.3
	Cultivar (C)	695.1	<0.0001		37.8	<0.0001	
	S x C	83.5	<0.0001		12.3	<0.0001	
Salinity tolerance index	Salinity	2083.4	<0.0001	7.9	294.6	<0.0001	13.4
	Cultivar	101.8	<0.0001		30.9	<0.0001	
	S x C	43.6	<0.0001		10.6	<0.0001	
Cotyledon fresh weight	Salinity	35.2	<0.0001	21.5	57.9	<0.0001	20.6
	Cultivar	55.1	<0.0001		41.0	<0.0001	
	S x C	1.6	<0.0001		2.1	<0.0001	
Seedling vigor index	Salinity	160.9	<0.0001	21.6	244.3	<0.0001	18.2
	Cultivar	56.5	<0.0001		97.2	<0.0001	
	S x C	5.9	<0.0001		12.4	<0.0001	

G_1_, Germination experiment-1 (12 cultivars); G_2_, Germination experiment-2 (subset of 6 cultivars selected based on G_1_ performance). CV (%), Coefficient of variation based on residual mean square error. All *p*-values < 0.0001 indicate statistically significant treatment effects. ANOVA includes main effects of salinity (S), cultivar (C), and their interaction (S × C).

#### 3.2.1 Effect of salinity on germination traits

**Figure 3:**
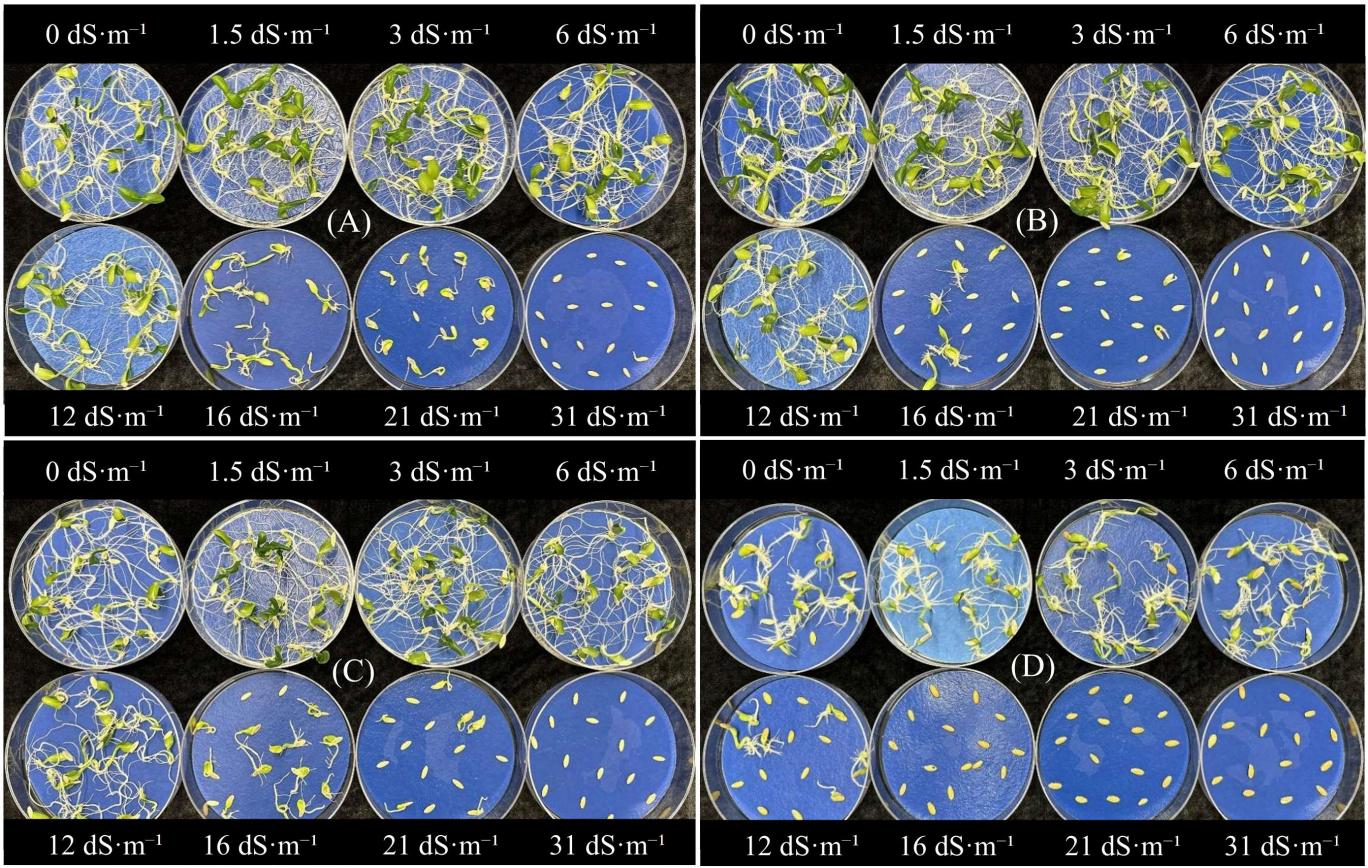
Effect of different brackish water treatments on seed germination and seedling growth in **(A)** Diva, **(B)** Katrina, **(C)** Suyo Long, and **(D)** Striped Armenian cucumber cultivars. Salinity treatments within each panel are arranged from left to right as follows: Top row – 0, 1.5, 3, and 6 dS·m^−^¹; Bottom row – 12, 16, 22, and 31 dS·m^−^¹.

Across salinity levels, the germination percentage gradually declined with increasing salinity (**Tables 2**, **3**). In G_1_, germination dropped from 92% at 0 dS·m^-1^ (control) to 38% at 21 dS·m^-1^, with complete failure at 31 dS·m^-1^. Similarly, in G_2_, germination declined from 98% at 0 dS·m^-1^ to 49% at 21 dS·m^-1^. These results indicate that increasing salinity levels severely restrict cucumber seed germination. This decline in germination may be attributed to osmotic stress, which limits water uptake during the critical imbibition phase of germination (Bakhshandeh et al., 2021; Al-Momany and Abu-Romman, 2023). Additionally, ion toxicity from excess Na^+^ and Cl⁻ disrupts membrane integrity and cellular function, further suppressing germination (Chen et al., 2020; Al-Momany and Abu-Romman, 2023). Several previous studies have reported similar reduction in germination with increasing salinity in cucumber (Chartzoulakis, 1992; Bakhshandeh et al., 2021). Similar trends have also been observed in other cucurbits such as pumpkin (*Cucurbita pepo* L.) (Irik and Bikmaz, 2024), Tunisian squash (*Cucurbita maxima* Duchesne) (Tarchoun et al., 2022), and muskmelon (Luis Castañares and Alberto Bouzo, 2019). These findings support the high sensitivity of cucumber, along with other cucurbit crops to salinity during the germination stage. The visual differences in germination and seedling development among cucumber cultivars across varying salinity levels are clearly illustrated in **Figure 3**. Salinity also significantly reduced the germination stress tolerance index, which reflects germination performance under stress relative to control (**Tables 2**, **3**). In G_1_, it declined from 100% at 0 dS·m^-1^ to 38% at 21 dS·m^-1^. Similarly, in G_2_, germination stress tolerance index declined from 100% at 6 dS·m^-1^ salinity to 50% at 22 dS·m^-1^, a 50% reduction. These results align with previous studies in cucumber (Marium et al., 2019) and squash (Tarchoun et al., 2022), confirming stress tolerance index as a reliable indicator of salinity tolerance during germination. Interestingly, cotyledon fresh weight increased slightly at mild salinity levels (1.5–6 dS·m^-1^) compared to 0 dS·m^-1^ in both experiments (**Tables 2**, **3**). This improvement may be partly attributed to the presence of beneficial nutrients in diluted brackish water, such as K^+^, Ca²^+^, and Mg²^+^ (**Supplementary Table 1**). These ions support membrane stability, enzymatic activity, and early seedling development (Mamedi et al., 2022; Niu et al., 2022). Additionally, low salinity may have triggered an osmotic priming effect, temporarily stimulating metabolic activity and enhancing seedling development (Nakaune et al., 2012; Matias et al., 2015). However, cotyledon fresh weight declined sharply at higher salinity levels (12–31 dS·m^-1^), indicating a negative impact on early seedling growth under increased salt stress. Similar results were reported in previous research on cucumber (Passam and Kakouriotis, 1994; Zhou et al., 2010; Chen et al., 2020) and pumpkin (Kusvuran et al., 2013; Irik and Bikmaz, 2024), where cotyledon fresh weight decreased with increasing salinity levels. Seedling vigor index showed a similar response, increasing slightly at 1.5–3 dS·m^-1^ but decreasing rapidly beyond 6 dS·m^-1^ in both experiments (**Tables 2**, **3**). According to previous studies on medicinal pumpkins (*Cucurbita pepo* subsp. *pepo* var. *styriaka*) (Farsaraei et al., 2021), regular pumpkins (Irik and Bikmaz, 2024), watermelon (*Citrullus lanatus* L.) (Li et al., 2023b), and melon (Oliveira et al., 2019), the seedling vigor index value dropped as salinity stress increased. These results confirm cucumber’s high sensitivity to salinity, with a critical threshold between 6 and 12 dS·m^-1^. This highlights the need for low-salinity irrigation during seed establishment and cultivar screening under mild stress to identify tolerant lines.

**Table 2:** Effect of salinity levels on germination percentage, salinity tolerance index, cotyledon fresh weight, and seedling vigor index in germination experiment-1 (G_1_).

Treatment	Germination percentage	Salinity tolerance index	Cotyledon fresh weight (g)	Seedling vigor index
Salinity (S)				
0 dS·m^-1^	92.1 ± 2.4 a^¥^	100.0 ± 0.0 a	0.18 ± 0.01 bc	6093.1 ± 393.3 b
1.5 dS·m^-1^	91.4 ± 2.0 a	100.8 ± 1.7 a	0.23 ± 0.01 a	8577.3 ± 659.5 a
3 dS·m^-1^	88.8 ± 2.4 b	97.0 ± 1.4 b	0.21 ± 0.01 a	8043.4 ± 609.1 a
6 dS·m^-1^	84.6 ± 3.4 c	89.5 ± 2.6 c	0.20 ± 0.01 ab	5929.2 ± 504.9 b
12 dS·m^-1^	77.6 ± 3.8 d	80.2 ± 3.9 d	0.15 ± 0.01 c	3353.3 ± 327.1 c
21 dS·m^-1^	37.6 ± 5.8 e	38.5 ± 5.9 e	0 ± 0.01 d	56.4 ± 27.4 d
31 dS·m^-1^	2.1 ± 0.7 f	0.0 ± 0 f	0.0 ± 0 e	0.0 ± 0 e
Cultivar (C)				
Cool Customer	63.1 ± 6.3 e	66.7 ± 6.7 e	0.20 ± 0.01 abc	6023.2 ± 745.0 b
Corinto	71.2 ± 8.2 d	72.4 ± 8.3 d	0.14 ± 0.01 de	4314.3 ± 535.6 bcd
Diva	86.9 ± 5.8 a	88.5 ± 5.9 a	0.24 ± 0.02 ab	7359.4 ± 822.7 b
H-19 Little Leaf	61.9 ± 7.7 e	61.9 ± 7.7 f	0.11 ± 0.01 c	2202.0 ± 308.9 c
Katrina	86.0 ± 5.9 a	86.0 ± 5.9 ab	0.26 ± 0.02 a	9880.1 ± 1208.7 a
Lemon	81.0 ± 6.5 b	83.8 ± 6.7 bc	0.19 ± 0.01 bcd	6908.5 ± 887.8 b
Marketmore76	78.8 ± 6.4 bc	81.6 ± 6.6 bc	0.19 ± 0.01 bcd	6214.4 ± 756.6 bc
Mexican Sour Gherkin	21.2 ± 4.0 g	53.6 ± 10.3 g	0 ± 0.00 f	0.0 ± 0.0 f
Picolino	68.8 ± 8.4 d	70.0 ± 8.6 d	0.18 ± 0.01 de	5546.6 ± 814.8 bc
Salt and Pepper	55.2 ± 6.8 f	66.3 ± 8.2 e	0.15 ± 0.01 cde	2812.2 ± 380.3 cd
Striped Armenian	62.4 ± 7.8 e	62.4 ± 7.8 f	0.16 ± 0.01 bcd	2744.2 ± 391.7 cd
Suyo Long	76.4 ± 6.5 c	77.8 ± 6.6 c	0.18 ± 0.01 cde	4759.3 ± 609.9 bcd

Values are expressed as mean ± standard error (n = 8). ^¥^Means within each column followed by the same letter are not significantly different according to Tukey’s HSD test at *p* < 0.05.

**Table 3:** Effect of salinity levels on germination percentage, salinity tolerance index, cotyledon fresh weight, and seedling vigor index in germination experiment-2 (G_2_).

Treatment	Germination percentage	Salinity tolerance index	Cotyledon fresh weight (g)	Seedling vigor index
Salinity (S)				
0 dS·m^-1^	97.5 ± 1.3 a^¥^	100.0 ± 0.0 a	0.20 ± 0.01 bc	8036.7 ± 686.8 c
1.5 dS·m^-1^	96.7 ± 1.1 ab	99.3 ± 1.0 ab	0.26 ± 0.01 a	10438.6 ± 799.4 a
3 dS·m^-1^	95.0 ± 1.7 ab	97.8 ± 2.2 ab	0.25 ± 0.02 ab	9206.8 ± 1014.1 b
6 dS·m^-1^	97.5 ± 1.0 a	100.4 ± 1.6 a	0.22 ± 0.01 abc	6848.8 ± 517.5 d
12 dS·m^-1^	88.9 ± 3.1 b	91.3 ± 3.2 b	0.18 ± 0.02 cd	3924.8 ± 381.7 e
16 dS·m^-1^	65.3 ± 7.4 c	67.8 ± 8.0 c	0.12 ± 0.01 de	777 ± 140.1 f
21 dS·m^-1^	48.9 ± 6.5 d	49.9 ± 6.6 d	0.07 ± 0.01 e	154.5 ± 42.2 f
31 dS·m^-1^	0.6 ± 0.4 e	0.6 ± 0.4 e	0.0 ± 0 f	0.0 ± 0 g
Cultivar (C)				
Diva	83.5 ± 5.5 a	85.0 ± 5.6 a	0.23 ± 0.02 ab	6198.5 ± 821.7 b
H-19 Little Leaf	69.8 ± 6.6 b	78.7 ± 7.5 bc	0.12 ± 0.01 c	3726.7 ± 421.2 c
Katrina	71.0 ± 6.8 b	71.0 ± 6.8 c	0.25 ± 0.02 a	9038.6 ± 1300.3 a
Lemon	82.3 ± 5.8 a	83.7 ± 5.8 ab	0.18 ± 0.01 bc	6548.5 ± 805.5 b
Striped Armenian	57.3 ± 7.7 c	58.2 ± 7.8 d	0.18 ± 0.02 bc	3358.0 ± 407.3 c
Suyo Long	78.8 ± 6.2 a	78.8 ± 6.2 ab	0.17 ± 0.01 bc	6087.6 ± 791.0 b

Values are expressed as mean ± standard error (n = 8). ^¥^Means within each column followed by the same letter are not significantly different according to Tukey’s HSD test at *p* < 0.05.

#### 3.2.2 Effect of cultivar selection on germination traits

Cucumber cultivars varied in their germination responses to salinity, reflecting genetic differences in salt tolerance (**Tables 2** and **3**). This variation was demonstrated in G_1_, where ‘Diva’ and ‘Katrina’ recorded the highest germination (87% and 86%, respectively), followed closely by ‘Lemon’ (81%) and ‘Marketmore 76’ (79%). In contrast, ‘Mexican Sour Gherkin’ showed the lowest germination (21%), while ‘Salt and Pepper’, ‘Striped Armenian’, and ‘H-19 Little Leaf’ also had relatively low germination (<65%). These differences were reflected in the germination stress tolerance index, where ‘Diva’ consistently ranked highest (88% in G_1_), followed by ‘Katrina’ and ‘Lemon’ (**Table 2**). Conversely, ‘Striped Armenian’ and ‘Mexican Sour Gherkin’ showed the lowest germination stress tolerance index values, indicating greater susceptibility to salt stress. Based on G_1_ performance, six cultivars were selected for G_2_, including the four most tolerant and two most sensitive lines. Similar results were observed in G_2_ as in G_1_. Cultivars ‘Diva’, ‘Katrina’, and ‘Lemon’ maintained high germination (84–82%) and germination stress tolerance index values (85–84%), indicating consistent tolerance (**Table 3**). In contrast, ‘Striped Armenian’ and ‘H-19 Little Leaf’ again performed poorly, with germination dropping to 57% and 70%, and germination stress tolerance index values of 58% and 79%, respectively. These similar results across both experiments show that cultivar responses were consistent and that the initial screening was effective.

Cotyledon fresh weight further differentiates cultivar performance (**Tables 2**, **3**). Cultivar ‘Katrina’ again exhibited the highest cotyledon fresh weight in both experiments, followed by ‘Diva’ and ‘Lemon’. In contrast, ‘Mexican Sour Gherkin’ showed no cotyledon development in G_1_. ‘Striped Armenian’ and ‘H-19 Little Leaf’ had the lowest measurable cotyledon fresh weight in G_2_. These cultivars showed up to 100% reduction in cotyledon weight compared to ‘Katrina’, highlighting their extreme salinity sensitivity. The seedling vigor index followed similar trends (**Table 2**). In G_1_, ‘Katrina’ had the highest seedling vigor index, surpassing Diva by 34% and Lemon by 78% (**Table 2**). Cultivars ‘Striped Armenian’, ‘Salt and Pepper’, and ‘H-19 Little Leaf’ had significantly lower seedling vigor index values of 72%, 71%, and 78% compared to ‘Katrina’, respectively. In G_2_, ‘Katrina’ again achieved the highest seedling vigor index, followed by ‘Diva’ and ‘Lemon’. Meanwhile, ‘Striped Armenian’ and ‘H-19 Little Leaf’ remained the lowest, consistent with their poor overall seedling performance. These cultivar-specific differences highlight the importance of genetic background in salinity tolerance during the germination stage (Tarchoun et al., 2022; Irik and Bikmaz, 2024). Such variation likely reflects underlying differences in ion regulation, osmolyte accumulation, and stress-induced signaling pathways (Du et al., 2010; Li et al., 2023b, 2023c). It is possible that because of the reasons stated above, cultivars ‘Diva’, ‘Katrina’, and ‘Lemon’ consistently performed well across multiple traits. While ‘Striped Armenian’ and ‘H-19 Little Leaf’ showed high sensitivity in germination experiments. Similar genotype-dependent responses have been reported in squash, where tolerant landraces maintained higher germination and seedling growth, while sensitive lines failed to germinate under high salinity (Tarchoun et al., 2022). Similar patterns were also seen in cucumber, where genotypes like ‘Valley’ and ‘HC-999’ outperformed others under salinity (Marium et al., 2019). Comparable findings in pumpkins also show that salinity stress sharply reduced germination, vigor, and root growth in sensitive cultivars (Irik and Bikmaz, 2024). These patterns across cucurbits, especially in cucumber, highlight the value of early germination screening for selecting salt-tolerant cultivars.

### 3.3 Seedling establishment under salinity stress

Based on germination performance, four cucumber cultivars— ‘Diva’, ‘Katrina’, ‘Striped Armenian’, and ‘Suyo Long’—were selected for further evaluation at the seedling stage under salinity stress. Salinity level and cucumber cultivar significantly affected seedling survival percentage, shoot fresh weight, shoot length, and SPAD. The interaction between salinity and cultivar was significant for all these parameters except survival percentage and SPAD (**Table 4**).

**Table 4:** Analysis of variance showing the effects of salinity, cultivar, and their interaction on survival percentage, shoot fresh weight, shoot length, and SPAD values in cucumber.

Treatment	Percentage survival	Shoot fresh weight (g)	Shoot length (cm)	SPAD
Salinity (S)				
0 dS·m^-1^	100.0 ± 0.0 a^¥^	37.8 ± 2.1 a	27.7 ± 2.6 b	47.9 ± 1.1 a
6 dS·m^-1^	100.0 ± 0.0 a	37.2 ± 3.3 a	39.8 ± 5.0 a	44.5 ± 1.2 ab
12 dS·m^-1^	87.5 ± 8.5 a	24.4 ± 2.6 b	23.3 ± 2.0 bc	47.4 ± 1.4 ab
14 dS·m^-1^	84.4 ± 8.8 a	16.1 ± 1.8 bc	17.3 ± 1.8 cd	43.3 ± 1.7 ab
16 dS·m^-1^	50.0 ± 12.9 b	9.2 ± 2.4 c	9.7 ± 2.0 d	47.9 ± 1.1 a
22 dS·m^-1^	0.0 ± 0.0 c	0.0 ± 0.0 d	0.0 ± 0.0 d	0.0 ± 0.0 c
F-value	37.6	49.6	46.3	3.9
*p*-value	<0.0001	<0.0001	<0.0001	0.0019
Cultivar (C)				
Diva	75.0 ± 9.0 a	15.2 ± 1.6 c	12.6 ± 1.0 c	49.8 ± 1.3 a
Katrina	72.9 ± 9.0 a	26.2 ± 2.6 b	21.4 ± 1.8 b	45.0 ± 1.2 bc
Striped Armenian	79.2 ± 8.5 a	41.9 ± 2.6 a	40.6 ± 4.2 a	45.3 ± 1.1 c
Suyo Long	54.2 ± 10.4 b	24.6 ± 2.6 bc	20.4 ± 2.8 b	42.9 ± 1.6 ab
F-value	4.5	63.5	61.2	6.6
*p*-value	0.0058	<0.0001	<0.0001	0.0001
S x C interaction				
F-value	1.1	2.2	5.7	3.5
*p*-value	0.4005	0.0169	<0.0001	0.6066
CV (%)	36.2	31.6	31.1	14.1

Data are presented as mean ± standard error (n = 8; 2 experimental runs × 4 replications). ^¥^Means within each column followed by the same letter are not significantly different according to Tukey’s HSD test at *p* < 0.05. SPAD: Soil-Plant Analyses Development (chlorophyll index). CV (%): Coefficient of variation based on residual mean square error.

#### 3.3.1 Effect of salinity on seedling establishment

Salinity levels had a pronounced impact on seedling survival, with 100% survival observed at 0 and 6 dS·m^-1^ (**Table 4**). However, survival declined slightly at 12 dS·m^-1^ (88%) and 14 dS·m^-1^ (84%). A sharp drop was observed at 16 dS·m^-1^ (50%), followed by complete mortality at 22 dS·m^-1^. This indicates a critical salinity threshold between 14–16 dS·m^-1^, beyond which seedling establishment is severely compromised. The decline in plant growth and survival with increasing salinity from 0 to 22 dS·m^-1^ is evident, as shown in **Figures 1C–F**. Consistent with survival trends, shoot fresh weight decreased significantly with increasing salinity (**Table 4**). At 6 dS·m^-1^, shoot fresh weight showed only a minor reduction (3%) compared to the control. However, more substantial declines were observed at 12 dS·m^-1^ (37%), 14 dS·m^-1^ (61%), and 16 dS·m^-1^ (76%). In contrast, shoot length increased by 43% at 6 dS·m^-1^ compared to the control, indicating a possible stimulatory effect of mild salinity. Beyond this level, shoot length declined progressively—by 18% at 12 dS·m^-1^, 43% at 14 dS·m^-1^, and 79% at 16 dS·m^-1^ relative to the control. Correspondingly, the shoot fresh weight stress tolerance index decreased as salinity increased (**Table 4**). It remained high at 6 dS·m^-1^ (97%), dropped to 63% at 12 dS·m^-1^, and decreased further to 39% and 24% at 14 and 16 dS·m^-1^, respectively (**Table 4**). SPAD readings, reflecting chlorophyll content, also declined significantly with increasing salinity (*p* = 0.0019). While values were relatively stable from 0 to 14 dS·m^-1^ (ranging from 48 to 44), a further drop to 42 was observed at 16 dS·m^-1^. No data were recorded at 22 dS·m^-1^ due to complete seedling mortality.

These results clearly show that increasing salinity levels negatively affect cucumber seedling growth and establishment. The gradual decline in survival, shoot biomass, and shoot length can be largely attributed to a combination of osmotic and ionic stresses (Lechno et al., 1997). High salt concentrations around the roots create osmotic stress, making it difficult for seedlings to absorb water (Yuan et al., 2016). This limits cell expansion, disrupts normal development, and reduces overall growth (Chartzoulakis, 1994). At moderate salinity, osmotic stress primarily hinders growth; however, as salinity rises, the excessive buildup of ions like Na^+^ and Cl^−^ results in ionic toxicity (Munns and Tester, 2008). For example, in our study, Na^+^ concentrations increased substantially under brackish water treatments (**Supplementary Table 1**). At 0 dS·m^-1^, Na^+^ was not detected. However, Na^+^ concentration increased to 881 ppm at 6 dS·m^-1^ and 1,741 ppm at 12 dS·m^-1^, reaching a peak of 3,561 ppm at 22 dS·m^-1^. Similarly, Cl^−^ concentrations increased from 3 ppm at 0 dS·m^-1^ to 1,555 ppm at 6 dS·m^-1^ and peaked at 5,410 ppm at 22 dS·m^-1^. Such elevated ion levels can disrupt cellular homeostasis, impair enzyme function, damage membranes, and ultimately lead to plant death (Chen et al., 2020). This pattern matches our results, where cucumber seedlings survived well up to 14 dS·m^-1^, but survival dropped to 50% at 16 dS·m^-1^ and reached 0% at 22 dS·m^-1^. In addition to osmotic and ionic stress, salinity may reduce shoot growth by disrupting uptake of essential nutrients like K^+^, Ca²^+^, and NO_3_^−^ (Kaya et al., 2007; Abbas et al., 2023). The deficiency of these nutrients, combined with excessive Na^+^ accumulation—particularly evident at 12 to 16 dS·m^-1^ salinity levels— reduces biomass accumulation, restricts shoot elongation and leads to a decline in SPAD readings. Similar results have been reported in other cucurbits like squash and melon, where salinity-induced nutrient imbalances reduced shoot biomass and growth (Romic et al., 2008; Uygur and Yetisir, 2009; Huang et al., 2010).

#### 3.3.2 Cultivar selection response to salinity stress at the seedling stage

**Figure 4:**
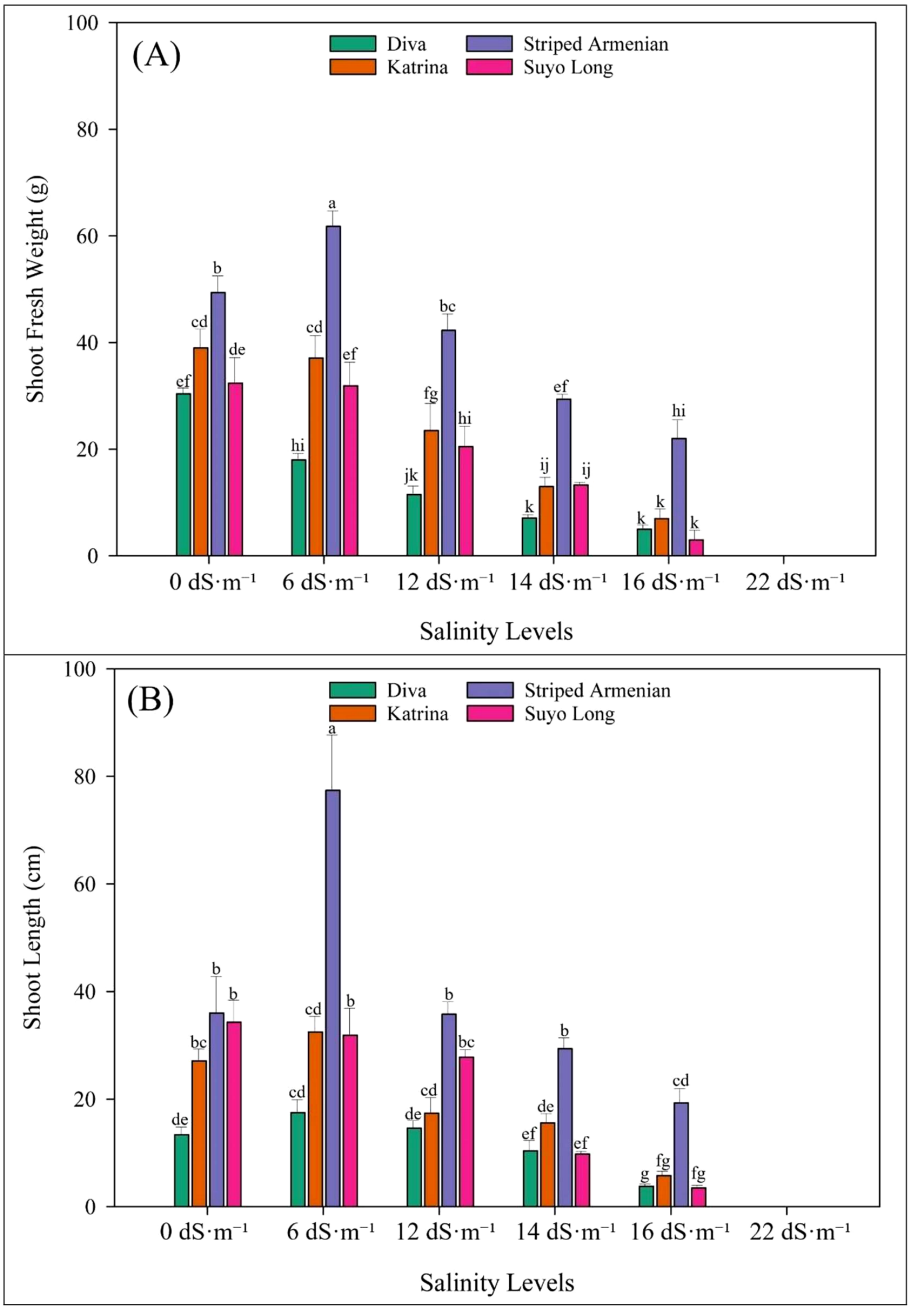
Effect of salinity levels on **(A)** shoot fresh weight and **(B)** shoot length of cucumber cultivars at seedling stage. Bars represent means, and error bars represent the standard error (SE, n=8, combined data from two experiments). Post-hoc tests were performed using LS means difference with Tukey's HSD analysis. In each graph, bars not sharing a common letter significantly differ at *p* < 0.05. No cultivars survived at 22 dS·m^-1^ salinity. Therefore, data is not shown for that treatment.

Among the tested cucumber cultivars, clear differences in seedling performance were observed under salinity stress (**Table 4**). Cultivar ‘Striped Armenian’ showed the highest survival rate (79%), followed closely by ‘Katrina’ (75%) and ‘Diva’ (73%), indicating better salt tolerance at the seedling establishment stage. In contrast, ‘Suyo Long’ recorded the lowest survival (54%), highlighting its greater sensitivity. In terms of shoot fresh weight, ‘Striped Armenian’ again outperformed the other cultivars, producing 72% and 73% more biomass compared to ‘Katrina’ and ‘Suyo Long’, respectively (**Table 4**). Cultivar ‘Diva’ exhibited the lowest shoot fresh weight, with a 65% reduction relative to cultivar ‘Striped Armenian’. Similar trends were observed for shoot length, where cultivar ‘Striped Armenian’ recorded the longest shoots—approximately 42% longer than those of cultivars ‘Katrina’ and ‘Suyo Long’ (**Table 4**). Conversely, ‘Katrina’ and ‘Suyo Long’ experienced moderate reductions in shoot length (48% and 51%, respectively). Cultivar ‘Diva’ showed the shortest shoots, with a 71% decrease compared to ‘Striped Armenian’. The interaction between salinity and cultivar was significant (*p* < 0.05) for both shoot fresh weight and shoot length (**Figure 4**). Cultivar ‘Striped Armenian’ consistently maintained the highest values across all salinity levels, from 6 to 16 dS·m^-1^. For example, its shoot fresh weight declined from 62 g at 6 dS·m^-1^ to 21 g at 16 dS·m^-1^, whereas ‘Diva’ dropped from 18 g to just 5 g over the same range. These results confirm ‘Striped Armenian’ superior to salinity tolerance and highlight the negative impact of increasing salt concentration on shoot growth, particularly in more sensitive cultivars like ‘Diva’ and ‘Suyo Long’. These results collectively indicate that ‘Striped Armenian’ consistently demonstrated superior performance across all measured traits at seedling stage. In contrast, ‘Suyo Long’ and ‘Diva’ were generally more sensitive to salinity, particularly in shoot-related parameters. The interaction between salinity levels and cultivars was significant (*p* < 0.05), revealing differential cultivar responses to salinity. ‘Striped Armenian’ consistently exhibited the highest shoot fresh weight across salinity levels, ranging from 62 g at 6 dS·m^-1^ to 21 g at 16 dS·m^-1^ (**Figure 4A**). In contrast, ‘Diva’ showed the lowest shoot fresh weight, declining from 18 g at 6 dS·m^-1^ to 5 g at 16 dS·m^-1^. For shoot length, ‘Striped Armenian’ maintained the highest values, reaching 36 cm at 6 dS·m^-1^ and decreasing to 16 cm at 16 dS·m^-1^ (**Figure 4B**). In contrast, ‘Diva’ had the shortest shoots, declining from 12 cm to 6 cm over the same salinity range. These results indicate a clear negative impact of increasing salinity on shoot growth, with ‘Striped Armenian’ demonstrating better tolerance compared to other cultivars. To better understand these performance differences under salinity, it is important to consider the physiological effects of salt stress on plant growth. Salinity stress is known to impair plant growth by inducing osmotic stress, ion toxicity, and nutrient imbalance, particularly at higher salt concentrations (Munns, 2002; Yuan et al., 2015). These physiological disruptions reduce water uptake and cell expansion, ultimately lowering survival and growth. In our study, cucumber seedlings showed full survival up to 6 dS·m^-1^, but survival declined sharply beyond 14 dS·m^-1^—indicating a threshold where stress effects intensify. This pattern aligns with earlier reports in cucumber (Chartzoulakis, 1994; Al-Sadi et al., 2010; Li et al., 2023b). Interestingly, ‘Striped Armenian’, which performed poorly at germination, emerged as the most resilient at the seedling stage—showing the highest survival, shoot length, and biomass. In contrast, ‘Diva’ and ‘Suyo Long’, which germinated well, were more sensitive under prolonged stress. This shift highlights the complexity of salinity tolerance and the importance of evaluating cultivars at multiple growth stages. The superior performance of ‘Striped Armenian’ may be linked to better mechanisms such as ion exclusion, osmotic adjustment, or cellular protection—traits that mitigate growth inhibition under stress (El-Shraiy et al., 2011; Abdel-Farid et al., 2020). These findings underscore the need for stage-specific screening and emphasize that early vigor doesn’t always translate to long-term tolerance.

#### 3.3.3 Stress indices and trait-based evaluation

Beyond growth parameters, shoot fresh weight-based stress indices offered further insights into cultivar responses to salinity. As salinity levels increased, stress intensity (SI) also increased progressively. It ranged from 0.02 at 6 dS·m^-1^ to 0.70 at 16 dS·m^-1^, reflecting increasing biomass reduction due to salinity stress (**Table 5**). The stress index values of cucumber cultivars are shown in **Table 5**. Across all salinity levels, the cultivar ‘Striped Armenian’ consistently outperformed the others. It recorded the highest shoot fresh weight under both control (*Y_p_*) and saline (*Y_s_*) conditions. As a result, it achieved the highest values for mean productivity (MP), geometric mean productivity (GMP), and stress tolerance index (STI). For instance, at 6 dS·m^-1^, stress tolerance index of ‘Striped Armenian’ was 2.1, while at 16 dS·m^-1^, it still maintained a relatively high-stress tolerance index value of 0.8, indicating strong tolerance. Additionally, it exhibited the lowest stress susceptibility index (SSI) and tolerance (TOL) values across salinity levels, suggesting better adaptability and lower yield loss under stress. In contrast, ‘Diva’ consistently exhibited the lowest values across most indices. At 16 dS·m^-1^, its shoot fresh weight dropped to 5.0 g, and the stress tolerance index fell to just 0.1. It also recorded the highest tolerance value (25.4) at 16 dS·m^-1^ salinity level, indicating its greater sensitivity to salinity stress. Similarly, ‘Suyo Long’ also showed weak performance under higher salinity. At 16 dS·m^-1^, it recorded the lowest geometric mean productivity of 9.9 and a stress tolerance index of 0.1 among all cultivars. However, cultivar ‘Katrina’ demonstrated intermediate performance. It maintained relatively stable biomass and index values at moderate salinity levels but experienced larger reductions at 16 dS·m^-1^. Overall, these results show that cultivar ‘Striped Armenian’ was the most salt-tolerant cultivar at the seedling stage, even though it performed poorly during germination. On the other hand, cultivar ‘Diva’ had good germination but was more sensitive to salinity at the seedling stage. This highlights the importance of evaluating cultivars at different growth stages and shows how stress indices can effectively identify salt-tolerant cultivars. This stage-specific response highlights the value of combining multiple stress indices for accurate salinity tolerance assessment. Indices such as the stress tolerance index and geometric mean productivity have proven effective in distinguishing tolerant lines in other cucurbits, including watermelon (Ekbic et al., 2017), bottle gourd (*Lagenaria siceraria*) (El-Shraiy et al., 2011), pumpkin (Horuz et al., 2022), squash (Tarchoun et al., 2022). Similarly, Yassin et al. (2019) validated these indices in wheat (*Triticum aestivum* L.) and noted the limited utility of stress susceptibility and tolerance indices—supporting our results.

**Table 5:** Stress tolerance indices based on shoot fresh weight of cucumber cultivars under varying salinity levels.

Salinity level	Cultivar	Y_p_	Y_s_	MP	STI	SSI	TOL	GMP
6 dS·m⁻¹	Diva	30.4	18.0	24.2	0.4	25.9	12.4	23.4
	Katrina	39.0	37.1	38.1	1.0	3.1	1.9	38.1
	Striped Armenian	49.4	61.8	55.6	2.1	-15.9	-12.4	55.2
	Suyo Long	32.4	31.9	32.1	0.7	1.0	0.5	32.1
	Mean	37.8	37.2	37.5	1.1	3.5	0.6	37.2
	Stress Intensity = 0.02							
12 dS·m⁻¹	Diva	30.4	11.5	20.9	0.2	1.8	18.9	18.7
	Katrina	39.0	23.5	31.3	0.6	1.1	15.5	30.3
	Striped Armenian	49.4	42.3	45.8	1.5	0.4	7.1	45.7
	Suyo Long	32.4	20.5	26.4	0.5	1.0	11.9	25.8
	Mean	37.8	24.4	31.1	0.7	1.1	13.3	30.1
	Stress Intensity = 0.35							
14 dS·m⁻¹	Diva	30.4	7.1	18.8	0.2	1.3	23.3	14.7
	Katrina	39.0	13.0	26.0	0.4	1.2	26.0	22.5
	Striped Armenian	49.4	29.4	39.4	1.0	0.7	20.0	38.1
	Suyo Long	32.4	13.3	22.8	0.3	1.0	19.1	20.7
	Mean	37.8	15.7	26.7	0.5	1.1	22.1	24.0
	Stress Intensity = 0.57							
16 dS·m⁻¹	Diva	30.4	5.0	17.7	0.1	1.2	25.4	12.3
	Katrina	39.0	7.0	23.0	0.2	1.2	32.0	16.5
	Striped Armenian	49.4	22.0	35.7	0.8	0.8	27.4	33.0
	Suyo Long	32.4	3.0	17.7	0.1	1.3	29.4	9.9
	Mean	37.8	9.3	23.5	0.3	1.1	28.5	17.9
	Stress Intensity = 0.70							

Y_p_ = Shoot fresh weight under normal conditions; Y_s_ = Shoot fresh weight under saline conditions; MP, Mean productivity; STI, Stress tolerance index; SSI, Stress susceptibility index; TOL, Tolerance; GMP, Geometric mean productivity. The 20 dS·m^-1^ salinity level was excluded from this analysis due to complete mortality of all cultivars.

The correlation analysis among stress indices is summarized in **Table 6**. Shoot fresh weight under non-saline (Yp) conditions showed strong positive correlations with geometric mean productivity (r = 0.78), mean productivity (r = 0.84), and stress tolerance index (r = 0.78). Similarly, shoot fresh weight under saline (Ys) conditions was highly correlated with mean productivity (r = 0.97), geometric mean productivity (r = 0.98), and stress tolerance index (r = 0.98). These associations suggest that cucumber cultivars that grew well under salinity stress also tended to grow well under normal conditions. Among all indices, the stress tolerance index had the strongest correlations with geometric mean productivity (r = 0.98) and mean productivity (r = 0.99). This reinforces its reliability as a key indicator for selecting salt-tolerant cultivars. On the other hand, the stress susceptibility index and tolerance values showed weak or negative relationships with most other traits, making them less useful in identifying truly tolerant cultivars. A similar trend was observed in watermelon, where cultivars with high tolerance and stress susceptibility index values performed poorly under salt stress (Ekbic et al., 2017). These results highlight stress tolerance index, mean productivity, and geometric mean productivity as reliable tools for identifying salt-tolerant cucumber cultivars at the seedling stage.

**Table 6:** Linear correlation among stress index attributes.

Trait	Yp	Ys	MP	STI	SSI	TOL
Ys	0.67 **					
MP	0.84 ***	0.97 ***				
STI	0.78 ***	0.98 ***	0.99 ***			
SSI	-0.45 ns	-0.42 ns	-0.46 ns	-0.49 ns		
TOL	-0.25 ns	-0.88 ***	-0.73 **	-0.79 ***	0.26 ns	
GMP	0.78 ***	0.98 ***	0.99 ***	0.98 ***	-0.40 ns	-0.78 ***

Y_p_ = Shoot fresh weight under normal conditions; Y_s_ = Shoot fresh weight under saline conditions; MP, Mean productivity; STI, Stress tolerance index; SSI, Stress susceptibility index; TOL, Tolerance; GMP, Geometric mean productivity. Significance levels: ***significant at the 0.1% level (*p* < 0.001); **significant at the 1% level (*p* < 0.01); ns, not significant (*p* ≥ 0.05).

### 3.4 Vegetative growth and physiological traits under salinity stress

Following germination stage screening, six cultivars with contrasting responses were selected to assess salinity tolerance at the vegetative stage. This allowed evaluation of their performance beyond early growth under continued salinity stress.

#### 3.4.1 Effect of salinity and cultivar selection on growth parameters

**Figure 5:**
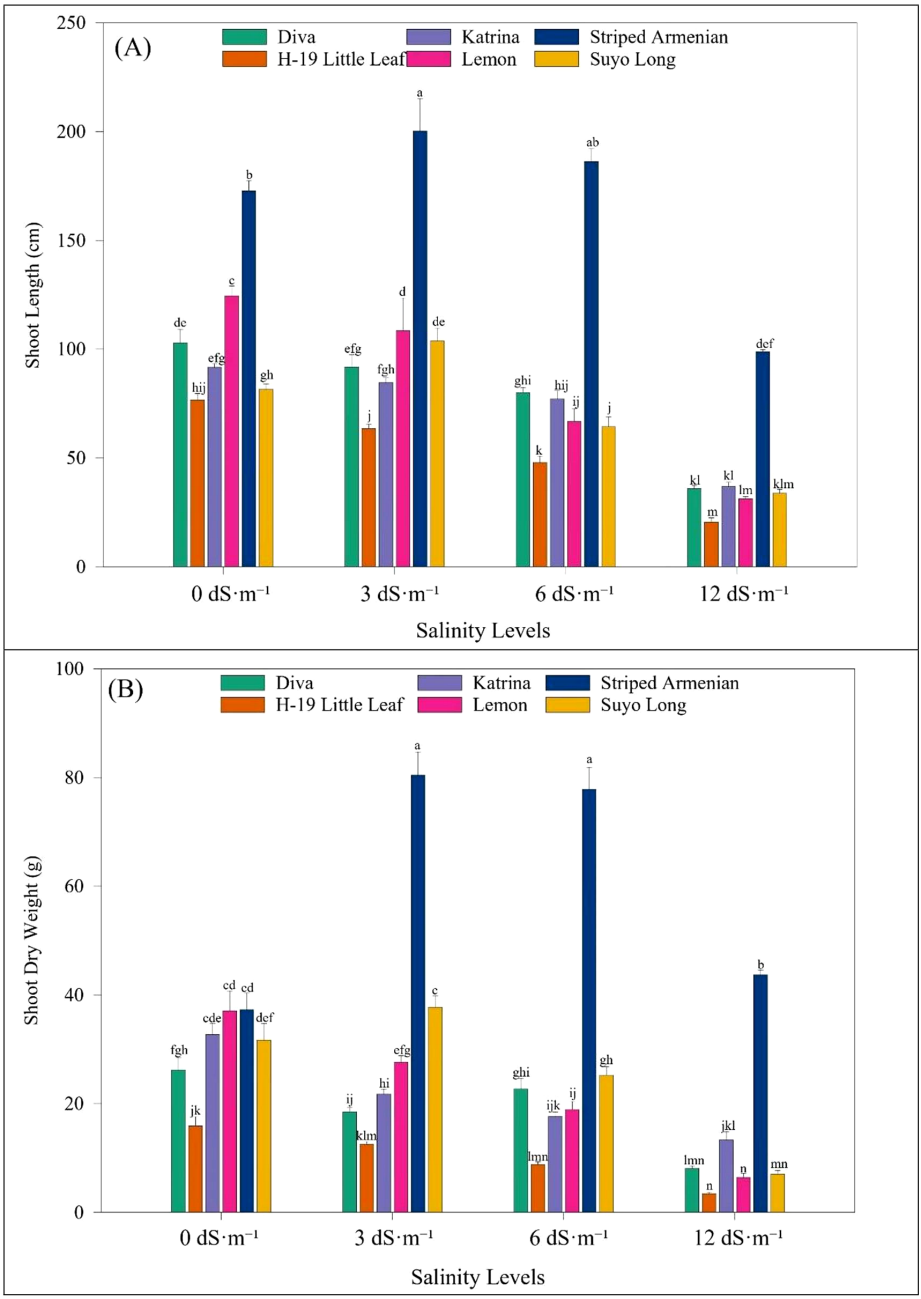
Effect of salinity levels on **(A)** shoot length and **(B)** shoot dry weight of cucumber cultivars at 21 days after salinity stress stage under hoophouse conditions. Bars represent means, and error bars represent the standard error (SE, n=8, combined data from two experiments). Post-hoc tests were performed using LS means difference with Tukey's HSD analysis. In each graph, bars not sharing a common letter significantly differ at *p* < 0.05.

Vegetative stage screening of six cucumber cultivars revealed significant effects of cultivar, salinity level, and their interaction on shoot length, shoot dry weight, and their respective stress tolerance indices (**Table 7**). As salinity increased, shoot growth and biomass declined across all cultivars. Shoot length decreased slightly by 0.4% at 3 dS·m^-1^, followed by sharper reductions of 20% at 6 dS·m^-1^ and 61% at 12 dS·m^-1^. Shoot dry weight showed a similar trend, decreasing by 39% at 3 dS·m^-1^, 52% at 6 dS·m^-1^, and 77% at 12 dS·m^-1^. Both shoot length and shoot dry weight stress tolerance indices declined with increasing salinity—dropping by 21% and 5% at 6 dS·m^-1^, and by 62% and 56% at 12 dS·m^-1^, respectively. Notably, some cultivars responded positively under mild stress. As seen during the seedling stage, ‘Striped Armenian’ continued to outperform other cultivars under salinity stress at the vegetative stage. It consistently showed the greatest shoot length and dry weight, standing out as the most resilient. In contrast, cultivars ‘H-19 Little Leaf’, ‘Diva’, and ‘Suyo Long’ experienced notable declines. Shoot length in these cultivars was reduced by 50–68%, and dry weight dropped by 58% to 83% compared to ‘Striped Armenian’. Similarly, stress tolerance indices were highest in ‘Striped Armenian’ and lowest in ‘Diva’ and ‘H-19 Little Leaf’, confirming their heightened sensitivity to salinity. The interaction between salinity levels and cultivars significantly affected shoot length and dry weight (**Figures 5A, B**). ‘Striped Armenian’ showed the highest tolerance, with a 16% increase in shoot length and a 35% increase in shoot dry weight at 3 dS·m^-1^. In contrast, ‘H-19 Little Leaf’ was the most sensitive, with shoot length reduced by 73% at 12 dS·m^-1^. ‘Lemon’ experienced the largest dry weight reduction (89%) at 12 dS·m^-1^, while other cultivars showed moderate declines.

**Table 7:** Cultivar and salinity levels affect vegetative growth and stress tolerance in cucumbers under hoop house conditions.

Treatment	Shoot length (cm)	Shoot dry weight (g)	Shoot length stress tolerant index	Shoot dry weight stress tolerant index
Salinity (S)				
0 dS·m^-1^	108.3 ± 5.2 a^¥^	30.1 ± 1.5 ab	100.0 ± 0.0 a	100.0 ± 0.0 a
3 dS·m^-1^	108.7 ± 7.0 a	33.1 ± 3.4 a	99.1 ± 3.5 a	98.9 ± 6.5 a
6 dS·m^-1^	87.1 ± 6.8 b	28.5 ± 3.4 b	77.5 ± 2.9 b	84.1 ± 6.8 a
12 dS·m^-1^	42.9 ± 3.8 c	13.7 ± 2.0 c	37.6 ± 1.7 c	36.3 ± 3.7 b
F-value	216.6	111.2	329.4	285.3
*p*-value	<0.0001	<0.0001	<0.0001	<0.0001
Cultivar (C)				
Diva	77.6 ± 5.0 bc	18.9 ± 1.4 c	75.5 ± 4.7 c	72.0 ± 5.1 bc
H-19 Little Leaf	52.1 ± 3.9 d	10.2 ± 0.9 d	68.0 ± 5.1 d	63.9 ± 5.3 bc
Katrina	72.6 ± 4.0 bc	21.3 ± 1.5 bc	79.2 ± 4.3 c	65.3 ± 4.2 bc
Lemon	82.8 ± 7.7 b	22.5 ± 2.3 bc	66.5 ± 6.1 d	60.7 ± 5.6 c
Striped Armenian	164.5 ± 7.7 a	59.8 ± 3.8 a	95.2 ± 4.3 a	136.9 ± 8.8 a
Suyo Long	70.8 ± 5.0 c	25.4 ± 2.3 b	86.9 ± 6.1 b	80.2 ± 6.8 b
F-value	235.1	291.6	31.7	176.4
*p*-value	<0.0001	<0.0001	<0.0001	<0.0001
S x C interaction				
F-value	7.4	27.7	6.9	31.4
*p*-value	<0.0001	<0.0001	<0.0001	<0.0001
CV (%)	16.8	19.1	14.2	15.3

^¥^Within a column, mean values (*n* = 8 plants; 2 experimental runs x 4 replications per experimental run) followed by a common letter are not significantly different from each other according to Tukey HSD (α = 0.05).

As observed during the germination and seedling stages, salinity continued to exert a clear negative effect on shoot growth and biomass even at the vegetative stage. Both shoot length and shoot dry weight declined significantly with increasing salinity, particularly at 6 and 12 dS·m^-1^—a trend also reported in melons and tomato cultivars (Botía et al., 1998; Sarabi et al., 2017; Agius et al., 2022). Interestingly, a slight increase in growth at 3 dS·m^-1^—most notably in ‘Striped Armenian’—suggests a mild stimulatory effect of low salinity (**Figures 2D, E**), possibly linked to enhanced ion uptake or metabolic activation (Kang et al., 2014; Sarabi et al., 2017). Similar growth improvements in the vegetative stage have been observed in other cucurbits, aligning with studies showing that mild salinity can enhance physiological functions (Chartzoulakis, 1992; Yetişir and Uygur, 2009; Tarchoun et al., 2022). These findings support the concept of hormesis, where exposure to low-level stress may trigger beneficial responses, particularly in ion-efficient or salt-tolerant cultivars (Giordano et al., 2021; Miceli et al., 2021). Despite its weak germination, ‘Striped Armenian’ exhibited strong shoot growth and salinity tolerance during later stages. Such contrasting cultivar responses are well documented across cucurbits. For instance, ‘Altinbas’ (tolerant) and ‘Citirex’ (sensitive) melon cultivars showed significant physiological divergence under salinity (Ulas et al., 2020). Similarly, tolerant pumpkin genotypes exhibited superior ion balance and stress indices compared to susceptible ones (Tarchoun et al., 2022; Irik and Bikmaz, 2024). Other studies also report clear salt stress contrasts between winter squash and pumpkin (Horuz et al., 2022) and between grafted cucumber lines using pumpkin rootstocks (Rouphael et al., 2012; Niu et al., 2017). These consistent trends across seedling and vegetative stages reinforce the robustness of ‘Striped Armenian’ under salinity stress and highlight the importance of multi-stage screening for selecting tolerant cultivars (**Figure 2F**).

Although ‘Striped Armenian’ performed poorly at germination under salinity, its superior growth at later stages suggests activation of post-germination tolerance mechanisms—such as ion compartmentalization, osmotic adjustment, or reactive oxygen species (ROS) detoxification—not yet functional during early development (Munns and Tester, 2008; Zhang and Shi, 2013; Shabala et al., 2015). These physiological processes are supported by molecular findings in cucurbits, where key regulatory genes such as *CsCBL4*, *CsCIPK6*, and *CsMAPK3/6* have been implicated in calcium signaling and MAPK pathways that regulate Na^+^/K^+^ balance and ROS scavenging (He et al., 2022; Wang et al., 2022). In salt-tolerant cucumber lines, enhanced activity of vacuolar Na^+^/H^+^ antiporters (e.g., NHX1) and increased expression of plasma membrane transporters (e.g., SOS1) contribute to effective Na^+^ sequestration and ionic homeostasis, while the upregulation of antioxidant enzymes helps mitigate oxidative damage during prolonged salt exposure (Huang et al., 2013; Li et al., 2023b; Amerian et al., 2024). Additionally, grafting cucumber onto salt-tolerant pumpkin or bottle gourd rootstocks has been shown to enhance ion exclusion and upregulate key salinity-responsive genes, further improving stress resilience (Rouphael et al., 2012; Peng et al., 2023a, 2023b). These molecular insights reinforce our phenotypic observations and suggest that the delayed but robust performance of ‘Striped Armenian’ likely reflects the onset of such downstream tolerance pathways during post-germination development. While this limits its use in direct seeding under saline conditions, it may be well suited for transplant-based systems or breeding efforts targeting long-term stress resilience (Ashraf and Foolad, 2013; Flowers and Colmer, 2015).

#### 3.4.2 Effect of salinity and cultivar selection on gas exchange

Gas exchange responses in cucumber cultivars were assessed at the vegetative stage, 7 and 21 days after salinity exposure. Salinity levels, cultivars, and their interactions had significant effects on all physiological traits (**Table 8**). Salinity had a clear and progressive impact on stomatal conductance. At 7 days after salinity treatment, stomatal conductance declined by 13% at 3 dS·m^-1^, 32% at 6 dS·m^-1^, and 54% at 12 dS·m^-1^. By 21 days, reductions were more severe—46% at 3 dS·m^-1^, 67% at 6 dS·m^-1^, and 86% at 12 dS·m^-1^ compared to the control. Similar trends were observed for transpiration rate, which decreased by 5%, 13%, and 24% at 3, 6, and 12 dS·m^-1^, respectively at 7 days. At 21 days, transpiration decreased by 28% at 6 dS·m^-1^ and 44% at 12 dS·m^-1^. Photosynthetic assimilation rate also declined with increasing salinity. At 6 dS·m^-1^, a 27% reduction was noted, and at 12 dS·m^-1^, photosynthesis dropped by 64%. Intercellular carbon dioxide concentration decreased with increasing salinity, particularly at 12 dS·m^-1^, where cultivars like ‘Diva’ and ‘Katrina’ showed pronounced reductions. Interestingly, intrinsic water use efficiency increased at 12 dS·m^-1^ salinity, indicating a potential adaptive response. Compared to lower salinity levels, 12 dS·m^-1^ led to the highest water use efficiency values for most cultivars at 21 days.

**Table 8:** Statistical significance of cultivar, salinity, and their interaction on gas exchange parameters at 7 and 21 days after salinity stress in cucumber under hoophouse conditions.

Treatment	Stomatal conductance (mol^.^m⁻²^.^s⁻¹)		Transpiration rate (mmol^.^m⁻²^.^s⁻¹)		Photosynthetic assimilation rate (µmol^.^m⁻²^.^s⁻¹)		Intercellular CO_2_ concentration (µmol^.^mol⁻¹)		Intrinsic water use efficiency (µmol^.^mol⁻¹)	
Salinity (S)	7^th^ day	21^st^ day	7^th^ day	21^st^ day	7^th^ day	21^st^ day	7^th^ day	21^st^ day	7^th^ day	21^st^ day
0 dS·m^-1^	0.9 a^¥^	1.1 a	12 a	8 a	30 a	25 a	343 a	375 a	37 c	25 d
3 dS·m^-1^	0.8 a	0.6 b	11 ab	6 b	28 ab	26 a	343 a	339 b	38 c	47 c
6 dS·m^-1^	0.6 b	0.3 c	10 b	6 b	27 b	19 b	315 b	308 c	56 a	67 b
12 dS·m^-1^	0.4 c	0.1 d	9 c	5 c	22 c	9 c	324 b	272 d	49 b	88 a
F-value	43.8	135.1	19.5	22.4	20.2	93.3	24.9	60.4	27.8	56.6
*p*-value	<0.0001	0.0002	<0.0001	0.0001	<0.0001	0.0008	0.0003	0.0001	<0.0001	<0.0001
Cultivar (C)										
Diva	0.5 c	0.4 b	9 c	5 c	23 c	18 b	318 b	303 b	56 a	71 a
H-19 Little Leaf	0.8 a	0.6 a	12 ab	6 abc	29 ab	24 a	335 a	342 a	42 b	43 b
Katrina	0.6 bc	0.5 ab	10 b	7 ab	26 bc	19 b	330 ab	305 b	47 b	67 a
Lemon	0.7 ab	0.5 ab	10 b	5 bc	26 bc	18 b	336 a	331 ab	43 b	54 ab
Striped Armenian	0.8 a	0.6 a	12 a	6 abc	30 a	21 ab	331 a	319 ab	43 b	60 ab
Suyo Long	0.7 ab	0.6 a	11 ab	8 a	27 abc	19 b	339 a	340 a	41 b	46 b
F-value	10.5	5.6	12.3	6.1	7.4	4.9	5.1	6.1	7.1	6.5
*p*-value	<0.0001	<0.0001	<0.0001	<0.0001	<0.0001	<0.0001	<0.0001	<0.0001	<0.0001	<0.0001
Interaction										
F-value	1.4	3.4	1.5	3.2	0.99	4.2	1.6	3.2	1.4	2.7
*p*-value	0.1372	0.0009	0.0939	0.0005	0.4618	<0.0001	0.0848	0.0005	0.1514	0.0025
CV (%)	28.1	31.3	18.5	21.1	19.1	19.5	5.6	8.4	27.2	30.9

^¥^Within a column, mean values (*n* = 8 plants; 2 experimental runs x 4 replications per experimental run) followed by a common letter are not significantly different from each other according to Tukey HSD (α = 0.05).

**Figure 6:**
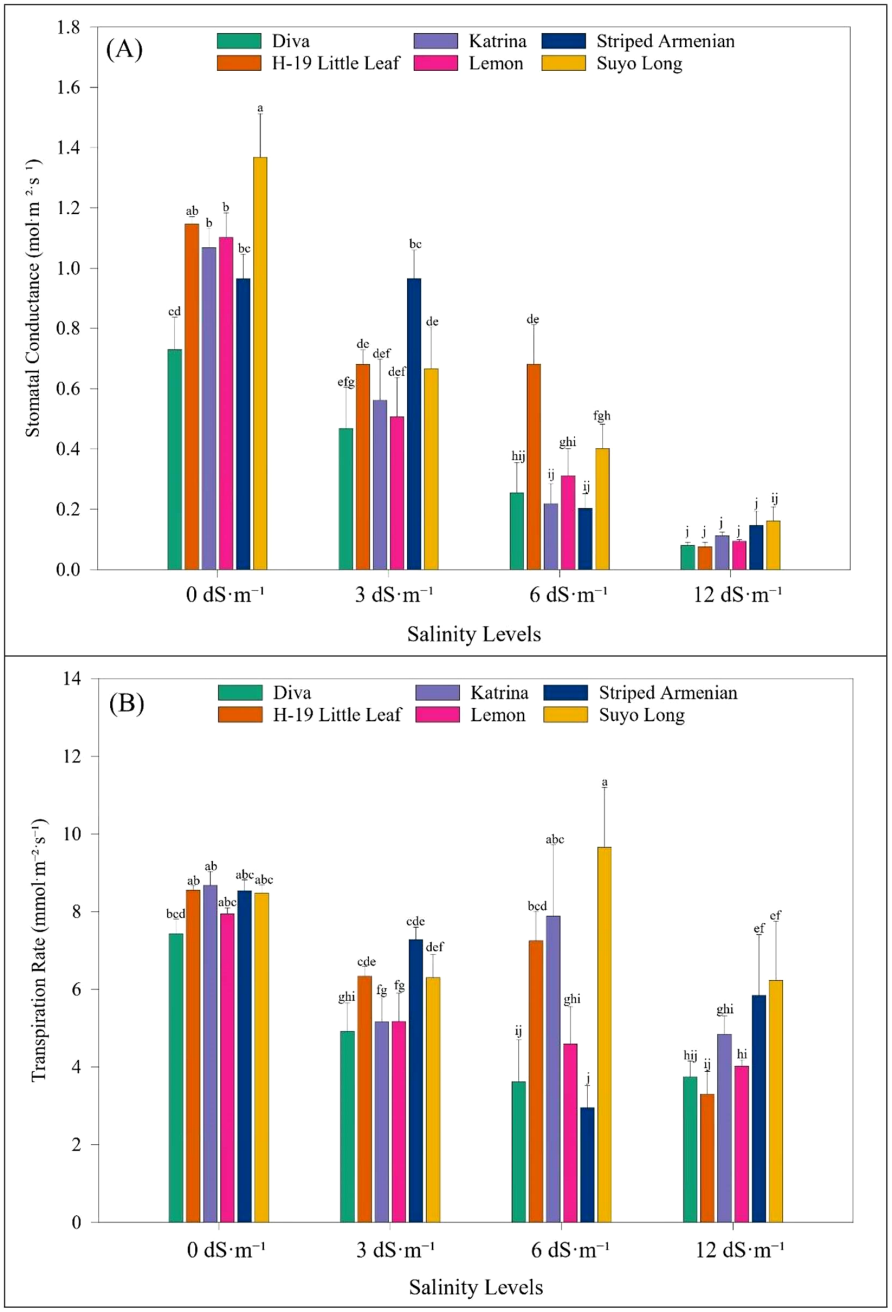
Effect of salinity levels on **(A)** stomatal conductance and **(B)** transpiration rate of cucumber cultivars 21 days after the salinity stress stage under hoophouse conditions. Bars represent means, and error bars represent the standard error (SE, n=8, combined data from two experiments). Post-hoc tests were performed using LS means difference with Tukey's HSD analysis. In each graph, bars not sharing a common letter significantly differ at *p* < 0.05.

**Figure 7:**
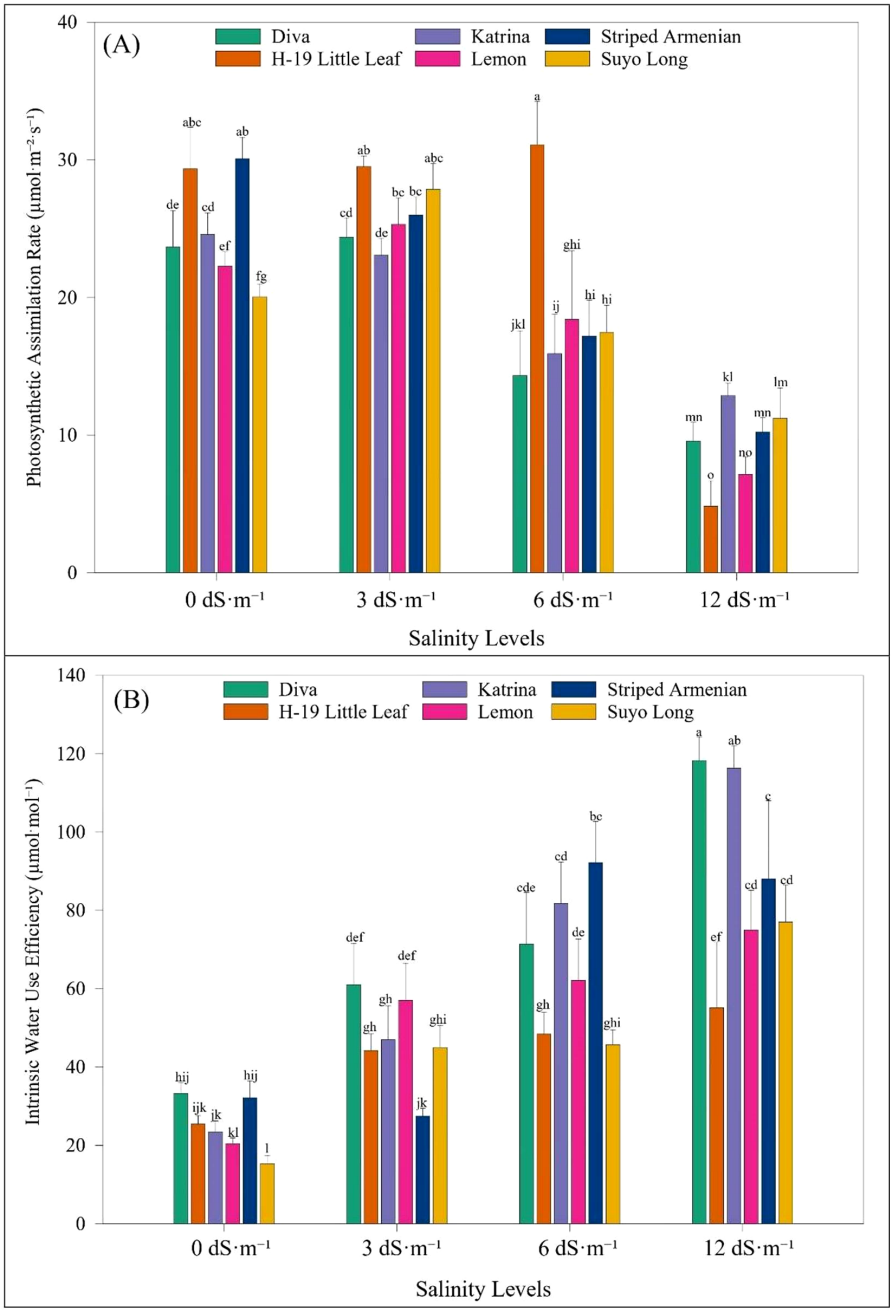
Effect of salinity levels on **(A)** photosynthetic assimilation rate and **(B)** intrinsic water use efficiency of cucumber cultivars 21 days after the salinity stress stage under hoophouse conditions. Bars represent means, and error bars represent the standard error (SE, n=8, combined data from two experiments). Post-hoc tests were performed using LS means difference with Tukey's HSD analysis. In each graph, bars not sharing a common letter significantly differ at *p* < 0.05.

Cultivar responses varied significantly. At 7 days after stress, ‘Striped Armenian’ recorded the highest stomatal conductance, followed by ‘H-19 Little Leaf’ and ‘Suyo Long’. ‘Diva’ showed the largest reduction (43%) relative to ‘Striped Armenian’. At 21 days, ‘Suyo Long’ had the highest stomatal conductance, while ‘Diva’ and ‘H-19 Little Leaf’ had the lowest values, with reductions up to 94% at 12 dS·m^-1^. For transpiration rate, ‘Striped Armenian’ again led at 7 days, while ‘Diva’ showed a 28% reduction. At 21 days, ‘Suyo Long’ exhibited the highest transpiration rate, while ‘Diva’ again had the lowest, with a 36% decline. The interaction between salinity and cultivar was significant, particularly at higher salinity levels. At 7 days after salinity exposure, ‘Striped Armenian’ had the highest stomatal conductance, while ‘Diva’ showed a 43% reduction. By 21 days, ‘Suyo Long’ exhibited partial recovery, maintaining higher conductance, whereas ‘Diva’ and ‘H-19 Little Leaf’ showed severe reductions of up to 94% at 12 dS·m^-1^ (**Figure 6A**). At 12 dS·m^-1^, reductions in transpiration ranged from 61% in ‘H-19 Little Leaf’ to 66% in ‘Striped Armenian’ (**Figure 6B**). Regarding photosynthetic assimilation, ‘Striped Armenian’ and ‘H-19 Little Leaf’ maintained relatively high rates at 7 days. At 21 days, ‘H-19 Little Leaf’ showed the highest photosynthetic rate, although differences with ‘Striped Armenian’ were not significant. Severe reductions were noted at 12 dS·m^-1^, especially in ‘Diva’ and ‘Lemon’, with over 60% decline. For intercellular carbon dioxide concentration, ‘Suyo Long’ maintained the highest levels across all salinity levels, while ‘Diva’ and ‘Katrina’ exhibited substantial decreases, particularly at 12 dS·m^-1^. In terms of water use efficiency, ‘Diva’ recorded the highest values under stress, followed by ‘Katrina’ and ‘Striped Armenian’. These cultivars showed increases of 67%, 73%, and 47%, respectively, at 12 dS·m^-1^ salinity (**Figure 7A**). In contrast, ‘Suyo Long’, ‘H-19 Little Leaf’, and ‘Lemon’ exhibited more modest gains or reductions, depending on salinity level. Overall, these results demonstrate the differential physiological responses of cucumber cultivars under salinity stress. ‘Striped Armenian’ consistently maintained higher physiological function under moderate stress. While ‘Diva’ and ‘H-19 Little Leaf’ showed early and severe declines, confirming their sensitivity.

The results from both time points—7 and 21 days after salinity stress—demonstrated that increasing salinity levels significantly reduced gas exchange parameters (Chartzoulakis, 1994; Rouphael et al., 2012). As salinity levels increased from 3 to 12 dS·m^-1^, all measured traits showed a steady decline. Photosynthetic assimilation and stomatal conductance were especially affected, with reductions exceeding 60% at the highest salinity level. This reduction is likely caused by the impact of high salt levels on stomatal function (Rouphael et al., 2012; Colla et al., 2013). Under salinity stress, plants limit stomatal opening and transpiration to conserve water and reduce ion toxicity (Yan et al., 2018; Horuz et al., 2022; Niu et al., 2022). Consequently, reduced stomatal conductance limits internal carbon dioxide availability, impairing photosynthetic activity (Horuz et al., 2022; Taha et al., 2022). This was evident in our study by the sharp declines in assimilation and transpiration, especially at higher salinity. The corresponding reduction in intercellular carbon dioxide concentration further indicates limited carbon fixation (Liu et al., 2012; Yang et al., 2015). These physiological constraints ultimately impair plant growth and water use efficiency, particularly in more sensitive cultivars (Yan et al., 2018; Horuz et al., 2022; Taha et al., 2022).

Although all cultivars showed reduced physiological performance with increasing salinity, the extent of decline varied among the cultivars evaluated, highlighting differences in tolerance. ‘Striped Armenian’ consistently maintained higher stomatal conductance and photosynthetic rates under moderate and severe salinity. This indicates better physiological regulation and stress adaptation during the vegetative stage (Elsheery et al., 2020; Parvathi et al., 2022). Despite its poor germination, its consistent performance at later stages reinforces the value of multi-stage screening in identifying tolerant cultivars (Marium et al., 2019). In contrast, ‘Diva’ exhibited sharp declines in all physiological parameters at higher salinity, despite its strong germination performance. Its inability to sustain gas exchange under prolonged stress contributed to reduced carbon assimilation and impaired growth during the vegetative stage (Liu et al., 2012; Yang et al., 2015; Kurtar et al., 2016; Ulas et al., 2020). Interestingly, ‘Suyo Long’ exhibited partial recovery in stomatal conductance and transpiration by 21 days under salinity stress. This transient improvement may be attributed to short-term osmotic adjustment or hormonal signaling rather than inherent stress tolerance (Zhu et al., 2019; Sarwar et al., 2021). Similarly, although sensitive during early growth, ‘H-19 Little Leaf’ maintained higher photosynthetic rates than ‘Diva’ at the vegetative stage, indicating possible delayed stress adaptation (Shireen et al., 2020; Peng et al., 2024).

Intrinsic water use efficiency increased in most cucumber cultivars under severe salinity, primarily due to reduced stomatal conductance. While this helps conserve water, it can also limit CO_2_ uptake, resulting in lower photosynthetic rates and reduced growth in sensitive cultivars like ‘Diva’ (Liu et al., 2012; Sarwar et al., 2021). Thus, although higher intrinsic water use efficiency may appear beneficial, it does not necessarily reflect true salinity tolerance (Modarelli et al., 2020). In our study, for example, ‘Katrina’ exhibited elevated intrinsic water use efficiency at 12 dS·m^-1^, but this was accompanied by significant reductions in shoot dry weight and photosynthetic rate (**Figure 7B**). This pattern suggests that increased intrinsic water use efficiency in ‘Katrina’ resulted more from stomatal restriction (i.e., a stress-avoidance mechanism) than efficient carbon assimilation. Similar findings have been reported in cucurbits such as muskmelon and grafted cucumber, where intrinsic water use efficiency gains under salinity were driven by reduced stomatal conductance rather than sustained productivity (Sarabi et al., 2019; Abbas et al., 2023). Therefore, intrinsic water use efficiency should be interpreted in the context of growth and photosynthetic performance to distinguish between physiological adaptation and stress avoidance. This nuance is important for accurately identifying salt-tolerant cultivars. Overall, evaluating traits such as gas exchange and intrinsic water use efficiency across time provides deeper insight into the physiological adjustments that underpin cultivar-specific salinity responses. These findings emphasize that long-term tolerance cannot be inferred from early-stage vigor alone and must be validated across developmental stages and trait types.

This study evaluated salinity responses only up to the vegetative stage. However, salinity stress during reproductive and fruiting stages can significantly impact yield and quality. Future experiments should extend into these later stages. A direct comparison between brackish water and pure NaCl treatments is also important. This will help separate the effects of osmotic stress from specific ion-toxicity. We screened only 12 commercial cucumber cultivars in this study. A larger screening that includes plant introductions, landraces, and wild accessions could identify additional sources of salt tolerance. To support phenotypic observations, ion accumulation (e.g., Na^+^, K^+^, Cl⁻) and nutrient analysis should be conducted. Biochemical profiling of antioxidant activity would provide further insight into stress responses. Finally, gene expression analysis of key transporters and regulatory genes would help validate the physiological mechanisms involved in salinity tolerance.

## 4 Conclusions

This study comprehensively evaluated the responses of cucumber cultivars to brackish water-induced salinity stress across three critical growth stages: germination, seedling establishment, and vegetative development. Increasing salinity consistently impaired plant performance, with significant reductions observed in germination percentage, seedling vigor, survival rate, biomass accumulation, and physiological functions such as stomatal conductance, transpiration, and photosynthesis. Interestingly, low salinity levels (1.5–3 dS·m^-1^) exerted a mild stimulatory effect—particularly during the germination and seedling stages—suggesting a hormetic response potentially linked to osmotic priming or enhanced nutrient uptake. This response was also detectable, though to a lesser extent, during the vegetative stage.

Cultivar responses were highly stage dependent. For instance, cultivars like ‘Diva’, ‘Katrina’, and ‘Lemon’ maintained high germination rates and vigor under mild salinity, while ‘Mexican Sour Gherkin’, ‘Striped Armenian’, and ‘H-19 Little Leaf’ showed early-stage sensitivity. Notably, ‘Striped Armenian’, despite poor germination, demonstrated superior tolerance at both the seedling and vegetative stages, highlighting the importance of stage-specific assessments. Conversely, ‘Diva’ and ‘Suyo Long’, which performed well during germination, exhibited reduced tolerance at later stages. In the vegetative phase, salinity stress significantly reduced shoot length, dry biomass, and gas exchange efficiency. Yet, ‘Striped Armenian’ consistently outperformed other cultivars, while ‘Diva’ and ‘H-19 Little Leaf’ showed substantial declines. ‘Suyo Long’ exhibited partial recovery in gas exchange traits by 21 days but remained limited in growth.

Based on our findings, we recommend the following stage-specific salinity thresholds for future screening and irrigation management. For the germination stage, salinity levels should be maintained at or below 6 dS·m^-1^ to ensure adequate seed emergence and early seedling vigor. During seedling establishment, a salinity range of 12 to 15 dS·m^-1^ is appropriate to evaluate cultivar survival and early biomass accumulation under moderate stress conditions. At the vegetative stage, salinity levels up to 12 dS·m^-1^ can be used to assess shoot growth and physiological responses such as gas exchange and water use efficiency. These thresholds provide a practical framework for stage-specific screening and can guide irrigation strategies in brackish water environments.

## Data Availability

The original contributions presented in the study are included in the article/**Supplementary Material**. Further inquiries can be directed to the corresponding authors.

## Author’s note

The use of trade, firm or corporation names in this publication is for the convenience of the reader. Such use does not constitute an official endorsement or approval by the United States Department of Agriculture or the Agricultural Research Service of any product or service to the exclusion of others that may also be suitable.
