# Supplementary material for: Growth stage-specific responses of cucumber to salinity stress: germination, seedling establishment, and vegetative development
Source: Front Plant Sci. 2025 Aug 13;16:1617809. doi: 10.3389/fpls.2025.1617809 (PMC12380902; doi:10.3389/fpls.2025.1617809)
Supplement: Supplementary file 1 [file Table1.docx]

# Supplementary Tables

**Supplementary Table 1.** The physicochemical properties and nutrient concentrations of brackish water solutions used in the salinity stress experiment across eight salinity levels (0% to 100%).

| **Parameter** | **0%** | **3.125%** | **6.25%** | **12.5%** | **25%** | **50%** | **75%** | **100%** |
| --- | --- | --- | --- | --- | --- | --- | --- | --- |
| EC (dS^.^m^-1^) | 0.009 | 1.6 | 3.052 | 6.049 | 11.43 | 21.76 | 31.38 | 40 |
| pH | 5.8 | 6.88 | 7.31 | 7.52 | 7.51 | 7.81 | 7.83 | 7.87 |
| mV | 77.3 | 11.3 | -13.9 | -26.2 | -25.5 | -43.1 | -44.6 | -46.6 |
|  | (*in ppm*) | | | | | | | |
| TDS | 5 | 799 | 1,496 | 2,961 | 5,600 | 10,660 | 15,370 | 19,980 |
| NO_3_-N | 0 | 0 | 0 | 0 | 0 | 0 | 0 | 0 |
| P | <0.02 | <0.02 | <0.02 | <0.02 | <0.02 | <0.02 | <0.02 | <0.02 |
| K | <0.1 | 9.12 | 17.46 | 37.81 | 77.97 | 157.3 | 239.8 | 330.2 |
| Ca | 0 | 7.75 | 16.04 | 35.45 | 70.56 | 121.3 | 172.98 | 220.25 |
| Mg | <0.01 | 30.06 | 57.91 | 118.54 | 225.07 | 425.23 | 616.95 | 807.15 |
| S | <0.02 | 21.89 | 40.28 | 83.4 | 163.84 | 306.86 | 440.16 | 571.66 |
| Zn | <0.01 | <0.01 | <0.01 | <0.01 | <0.01 | <0.01 | <0.01 | <0.01 |
| Cu | <0.01 | <0.01 | <0.01 | <0.01 | <0.01 | <0.01 | <0.01 | <0.01 |
| Mn | <0.01 | <0.01 | <0.01 | <0.01 | <0.01 | <0.01 | <0.01 | <0.01 |
| Fe | 0.02 | 0.02 | 0.022 | 0.027 | 0.034 | 0.041 | 0.047 | 0.051 |
| B | <0.01 | 0.09 | 0.19 | 0.39 | 0.76 | 1.45 | 2.03 | 2.55 |
| Na | <0.0 | 245 | 460 | 881 | 1,741 | 3,561 | 5,375 | 7,101 |
| Cl | 3 | 453 | 837 | 1,555 | 2,645 | 5,410 | 7,330 | 9,250 |
| As | <0.0 | <0.0 | <0.0 | <0.0 | <0.0 | <0.0 | <0.0 | <0.0 |
| Cd | <0.0 | <0.0 | <0.0 | <0.0 | <0.0 | <0.0 | <0.0 | <0.0 |
| Cr | <0.0 | <0.0 | <0.0 | <0.0 | <0.0 | <0.0 | <0.0 | <0.0 |
| Mo | <0.0 | <0.0 | <0.0 | <0.0 | <0.0 | <0.0 | <0.0 | <0.0 |
| Ni | <0.0 | <0.0 | <0.0 | <0.0 | <0.0 | <0.0 | <0.0 | <0.0 |
| Pb | <0.0 | <0.0 | <0.0 | <0.0 | <0.0 | <0.0 | <0.0 | <0.0 |
| Se | <0.0 | <0.0 | <0.0 | <0.0 | <0.0 | <0.0 | <0.0 | <0.0 |

EC = Electrical conductivity; mV = Redox potential; TDS = Total dissolved solids.

< indicates below quantifiable level of detection.
